# An Optimized Transformation System and Functional Test of *CYC*-Like TCP Gene *CpCYC* in *Chirita pumila* (Gesneriaceae)

**DOI:** 10.3390/ijms22094544

**Published:** 2021-04-27

**Authors:** Jing Liu, Juan-Juan Wang, Jie Wu, Yang Wang, Qi Liu, Fang-Pu Liu, Xia Yang, Yin-Zheng Wang

**Affiliations:** 1 State Key Laboratory of Systematic and Evolutionary Botany, Institute of Botany, Chinese Academy of Sciences, Beijing 100093, China; liujing2010@ibcas.ac.cn (J.L.); jwang2@mpipz.mpg.de (J.-J.W.); daisywujie@mail.tsinghua.edu.cn (J.W.); wangyang2017@ibcas.ac.cn (Y.W.); liuqi2017@ibcas.ac.cn (Q.L.); liufangpu@ibcas.ac.cn (F.-P.L.); 2 University of Chinese Academy of Sciences, Beijing 101408, China

**Keywords:** *Chirita pumila*, *CpCYC*, evo-devo, functional test, genetic transformation, Gesneriaceae, a new model

## Abstract

The development of an ideal model plant located at a key phylogenetic node is critically important to advance functional and regulatory studies of key regulatory genes in the evolutionary developmental (evo-devo) biology field. In this study, we selected *Chirita pumila* in the family Gesneriaceae, a basal group in Lamiales, as a model plant to optimize its genetic transformation system established previously by us through investigating a series of factors and further conduct functional test of the *CYC*-like floral symmetry gene *CpCYC*. By transforming a RNAi:CpCYC vector, we successfully achieved the desired phenotypes of upright actinomorphic flowers, which suggest that *CpCYC* actually determines the establishment of floral zygomorphy and the horizontal orientation of flowers in *C*. *pumila*. We also confirmed the activities of *CpCYC* promoter in dorsal petals, dorsal/lateral staminodes, as well as the pedicel by transferring a *CpCYC* promoter:GUS vector into *C*. *pumila*. Furthermore, we testified the availability of a transient gene expression system using *C*. *pumila* mesophyll protoplasts. The improved transformation system together with the inherent biological features would make *C. pumila* an attractive new model in functional and regulatory studies for a broad range of evo-devo issues.

## 1. Introduction

There are an estimated 352,000 species of flowering plants or angiosperms, much more than all other land plants that count no more than 35,000 species [1]. The dominance of angiosperms might be partly due to the complex flower organization, a highly sophisticated system for insect pollination [2]. Therefore, understanding the exciting diversity of floral architecture has become a very attractive subject for evolutionary developmental (evo-devo) biologists [3]. The organ types determined by organ identity genes functioning in frame of the ABC model are the basis of final floral form. According to the classical ABC model, the identities of sepals, petals, stamens, and carpels within a flower are, respectively, specified by A-function, A- and B-functions, B- and C-functions, and C-function [4,5]. The debates about the A-function defined in the classical ABC model have become conspicuous [3,6]. Therefore, it is urgent to conduct systematic comparative analyses of A-function genes in more species with key phylogenetic position outside the classical model plants [3,6,7].

Floral zygomorphy has been widely considered to be one of the key innovations relating to the explosive radiation of angiosperms [8,9,10]. The molecular basis for floral zygomorphy was first uncovered in *Antirrhinum*, in which two TCP genes - *CYCLOIDEA* (*CYC*) and *DICHOTOMA* (*DICH*) - play a key role in patterning floral zygomorphy via specifically regulating the development of dorsal petals and stamens [11,12]. Later studies revealed that repeated gains of auto-regulatory loops in *CYC*-like genes allow them to be recurrently recruited in different clades of angiosperms [13], in which widely diverse modifications further give rise to adaptive radiation of zygomorphic groups [13,14,15,16,17,18,19,20]. However, there is still little knowledge about how the divergent expression patterns of *CYC*-like genes are controlled by upstream *cis*-regulatory elements and *trans*-acting factors and how they in turn regulate their downstream targets [21].

*Agrobacterium*-mediated transformation is a widely used and powerful tool for the analysis of gene functions and their regulatory network. Limited transformation capability is a significant barrier to our understanding of these topics. At present, evo-devo researches, functional assays, rapid determination of subcellular protein localization, as well as protein–DNA and protein–protein interactions are usually conducted exogenously in proficient model systems because of the difficulty in carrying out such experiments endogenously in many species. For example, the genetic transformation is still difficult even in some classical model plants or important model crops, such as *Antirrhinum*, *Aquilegia*, and *Glycine max* (Table 1) [22,23,24,25,26]. It has been increasingly recognized that the lack of functional analyses based on stable genetic transformation has greatly restricted our knowledge about floral development in evo-devo points of view [3,27]. Therefore, it is urgent to develop more new models with key phylogenetic location outside the classical model plants. Organisms with efficient transformation systems would have a prominent place in this new field, especially in floral symmetry, as snapdragon falls down due to its difficulty in genetic transformation.

**Figure 1:**
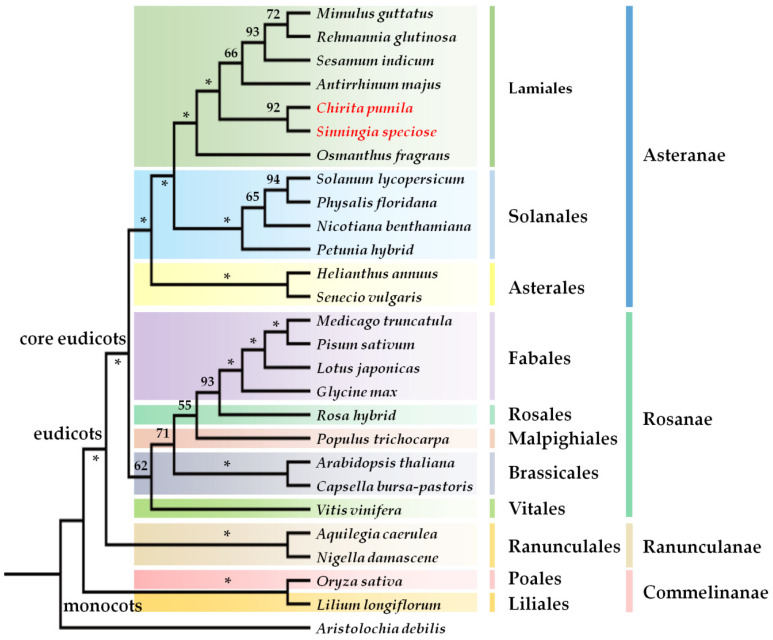
The strict consensus tree showing relative positions of major model systems. The tree is generated from datasets of combined *rbcl*, *matk* and *ndhf* data. Bootstrap values are labeled above the branches. The asterisk indicates 100% support. The species in red font indicate two representatives of Gesneriaceae.

Lamiales is a major angiosperm clade predominant with zygomorphic flowers that are believed to be the ancestral state in this order [9,42,43]. Gesneriaceae, a basal group of Lamiales, represents an important phylogenetic node relating to some key evolutionary questions in Lamiales, even in Asterids (Figure 1), and is rich in various types of floral symmetry [9]. The key phylogenetic location allows the development of a new model in this family to address a broad range of evo-devo questions, especially in the field of floral evolution. *Chirita pumila*, as a representative of Gesneriaceae, has many advantages common to model plants and its whole genome sequencing is on-going in our group. *Chirita pumila* produces delicate zygomorphic flowers, with a bilabiate corolla and two ventral stamens with both dorsal and lateral stamens aborted. The zygomorphy is also reflected in the corolla tube where a ridge structure and a yellow spot label the dorsal and ventral identities, respectively, as well as in the stigma with two upper lobes almost sterile and the lower ones strongly enlarged [44]. What is even more exciting is that *C*. *pumila* shares a series of common biological features with classical model plants. *Chirita pumila* is diploid (2 *n* = 8) and has a small genome size that is estimated to be 798.7 Mbp [29]. In contrast to some tall and large model plants (such as *Oryza sativa*, *Glycine max* and *Populus trichocarpa*), *C*. *pumila* exhibits a compact plant architecture like that of *Arabidopsis* and *Lotus japonicus* (Table 1). This makes *C*. *pumila* well suited to being cultivated in growth chambers with high density until flowering. The plant of *C*. *pumila* can grow healthily under 23–26 °C; and a broad range of light intensity. The lifecycle of *C*. *pumila* can be finished within five months in the chamber. One capsule of *C*. *pumila* yields more than one thousand tiny seeds, which indicates its high fecundity. The large zygomorphic flowers of *C*. *pumila* reaching to 3–4 cm in length and 2–3 cm in width makes it easy for us to identify mutants and carry out hand-pollination. The small plant size, short generation time, high fecundity, and small genome size of *C*. *pumila* are common features of most model plants, such as *Arabidopsis*, *Medicago*, and *Mimulus*. *Chirita pumila* even has a series of advantages over some classical model plants, such as, *Petunia*, *Helianthus*, and *Nicotiana* with large genome and plant size (Table 1). In consideration of these biological and genetic advantages, we developed an initial *Agrobacterium*-mediated genetic transformation system in *C. pumila* seven years ago [29]. However, it is necessary to further improve some essential aspects in efficiency and stability of the transformation system in *C. pumila* for the urgent need of large-scale functional studies in evo-devo. These aspects include *Agrobacterium* strains, MS strength, sucrose concentration, selection reagents for positive transformed cells and antibiotics used to eliminate *Agrobacterium* from plant tissues in regeneration media, etc. [45].

In this study, we first comprehensively optimized the culture conditions of *C. pumila* by adjusting several parameters that influence seed germination, seedling growth, and tissue culture. We then greatly improved the efficiency and stability of the transformation system. In addition, we investigated the transient expression assay system of *C. pumila* mesophyll protoplast used in subcellular protein localization and protein-protein interaction analyses. Furthermore, we successfully carried out the function study of a *CYC*-like gene, *CpCYC*, by RNA interference (RNAi) technology, which clearly demonstrates the function of *CpCYC* actually controlling the development of dorsal floral organs and the orientation of flowers in *C. pumila*. Last, we testified the ability of this transformation system in studying regulatory mechanisms of gene expression by transferring a *CpCYC* promoter:GUS reporter vector. The GUS signals in dorsal petals, dorsal and lateral staminodes, as well as the pedicel are consistent with the functional domains of *CpCYC* illustrated by the floral phenotypes of *RNAi:CpCYC* mutant flowers. Our results indicate that *C. pumila* is an ideal emerging model plant for a broad range of issues in evo-devo biology.

## 2. Results

### 2.1. Optimization of the Media Used for Seed Germination, Tissue Culture and Seedling Growth

Availability of enough and healthy explants is a prerequisite for an efficient transgenic system. To accelerate seed germination, we examined the germination rate of seeds on different germination media (SGM; Table 2). On SGM-IV (1/2 MS medium supplemented with 1% sucrose), radicle began to elongate after five-days of sowing, and green cotyledons could be clearly observed with naked eyes on the seventh day. By contrast, 1/2 MS media supplemented with 2% and 3% sucrose (SGM-V and SGM-VI) delayed seed germination for two and three days, respectively. On the full-strength MS medium with 1% sucrose (SGM-I), seeds germinated one day later than that germinated on the optimal medium SGM-IV. Furthermore, the full-strength MS media supplemented with 2% and 3% sucrose (SGM-II and SGM-III) delayed seed germination dramatically, with 12 and 17 days, respectively, required for seed germination (Table S1). We conclude that 1/2 MS with 1% sucrose was the optimal medium for seed germination, although all media gave rise to 100% seed germination rate (Table S1).

**Figure 2:**
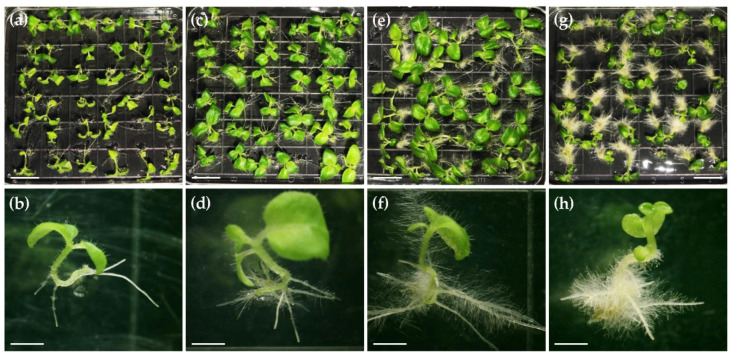
Effects of NAA on seedling growth. (**a**,**b**) Seedlings grown on medium without external phytohormone. (**c**,**d**) Seedlings grown on medium supplemented with 0.02 mg/L NAA. (**e**,**f**) Seedlings grown on medium supplemented with 0.05 mg/L NAA. (**g**,**h**) Seedlings grown on medium supplemented with 0.1 mg/L NAA. Scale bars: 1 cm in panels (**a**,**c**,**e**,**g**); 0.2 cm in panels (**b**,**d**,**f**,**h**).

We then checked whether α-naphthalene acetic acid (NAA), a kind of plant growth regulator, has a positive effect on seed germination in *C. pumila* using the optimal seed germination medium SGM-IV supplemented with NAA of different concentrations. Although having no effect on accelerating seed germination, NAA was found to have an obvious effect on the seedling elongation and hairy roots development just after germination (Figure 2). We found that 0.02 mg/L NAA accelerated the outgrowth of hairy roots and therefore promoted the upright growth of seedlings (Figure 2a–d). However, when we further increased the NAA dosage to 0.05 and 0.1 mg/L, seedling growth was significantly repressed while excessive hairy roots produced (Figure 2e–h). Taken together, 1/2 MS medium with 1% sucrose and 0.02 mg/L NAA was used for seed germination in following experiments.

**Figure 3:**
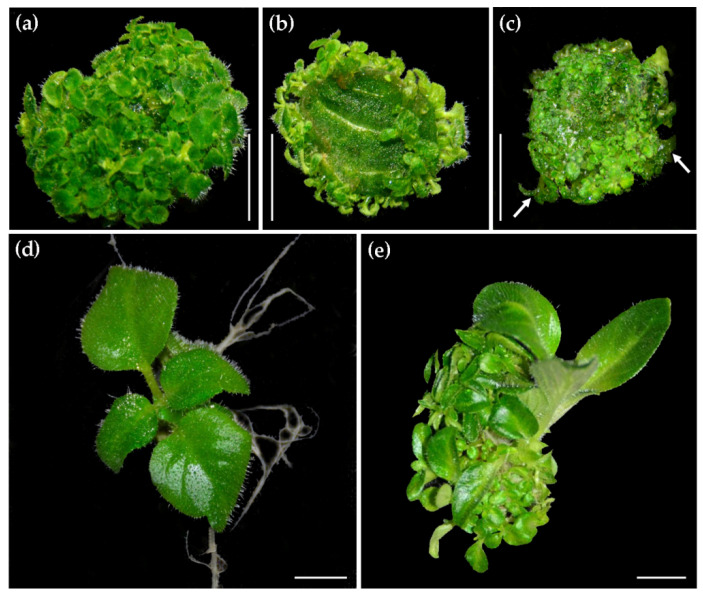
Effect of placing orientation of leaf explants on shoot regeneration. (**a**) The adaxial side of leaf explants was placed upward. (**b**) The abaxial side of leaf explants was placed upward. (**c**) The adaxial view of explants cultured with abaxial surface upward. (**d**) A seedling grown on the medium without 6-BA and NAA. (**e**) A seedling grown on the medium containing 0.5 mg/L 6-BA and 0.1 mg/L NAA. Scale bars: 0.5 cm.

We previously reported a high-efficiency adventitious shoot induction method by culturing *C. pumila* leaf explants on TCM-III (Table 2) [29]. However, under this condition, excessive regeneration of adventitious buds severely affects their growth. As a first step to solve this problem, we checked whether the placing orientation of leaf explants affects adventitious bud induction rate and shoot growth state. Our results showed that adventitious buds always generated from the adaxial side of leaf explants, regardless of the placing orientation of explants (with either adaxial or abaxial surfaces upward) on the medium (Figure 3a,b). In addition, when explants were placed on medium with their abaxial surfaces upward, induced shoots were usually vitrified and deformed (Figure 3c). Therefore, to obtain more and healthy adventitious shoots, leaf explants should be cultured with their adaxial surfaces upward. To detect the effect of 6-benzyl amino purine (6-BA) and NAA on shoot elongation, we compared the states of seedling growth on medium with or without 6-BA and NAA. We found that the persistent existence of 6-BA and NAA produced a great number of adventitious buds around main shoots and thereafter inhibited their elongation (Figure 3d,e). Therefore, 6-BA and NAA should be removed at shoot elongation stage.

To further overcome the problem of excessive adventitious bud induction, we investigated the effect of different tissue culture media (TCM; Table 2) on shoot regeneration. Three weeks after induction, all experimental groups gave birth to 100% shoot induction rate. Both MS and 1/2 MS media containing 1% sucrose (TCM-I and TCM-IV) generated relatively fewer and larger adventitious buds (Table 3; Figure S1a,d). Nevertheless, MS medium with 1% sucrose (TCM-I) usually generated vitrified shoots (Table 3; Figure S1a). By contrast, when 2% or 3% sucrose was used, adventitious shoots were small and clustered under both full- and half-strength MS conditions (Table 3; Figure S1b,c,e,f). Thus, we take the media (TCM-IV) containing 1/2 MS and 1% sucrose as the most suitable medium for adventitious bud induction.

To select an optimal medium for shoot elongation and rooting, the adventitious shoots from the optimal adventitious bud induction medium TCM-IV were further sub-cultured onto SGM-I, SGM-II, SGM-III, SGM-IV, SGM-V, and SGM-VI. The results showed that adventitious shoots on SGM-V grew fastest with well-developed leaves and root systems consistent with the elongation situation of seedlings germinated from seeds (Table 3; Figure S1g–l). Taken together, TCM-IV containing 1/2 MS salt and 1% sucrose was optimal for adventitious shoot induction, while 1/2 MS medium containing 2% sucrose was most suitable for shoot elongation and rooting.

### 2.2. Optimization of the Genetic Transformation System in C. pumila

**Figure 4:**
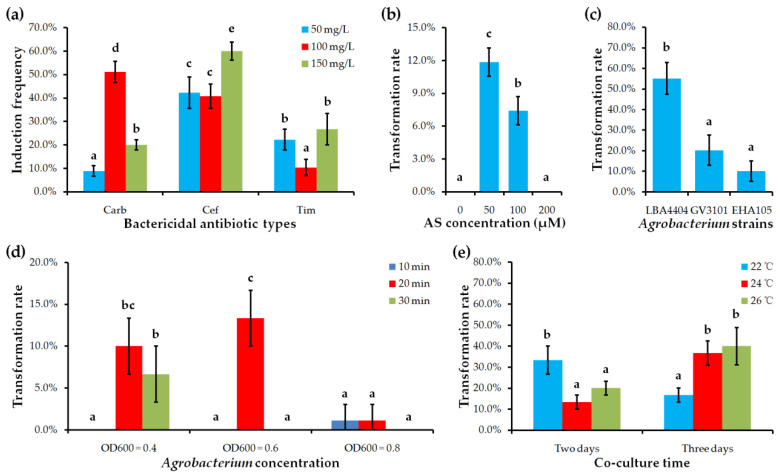
Effect of different factors on transformation rate or shoot induction frequency. (**a**) Bactericidal antibiotic types and their concentrations. (**b**) Acetosyringone (AS) concentration. (**c**) *Agrobacterium* strains. (**d**) *Agrobacterium* concentration and incubation time. (**e**) Co-culture time and temperature. Values shown (mean ± standard deviation) are the average of three replicates with each replicate containing 30–45 explants. The difference significance was tested using the LSD test (*p* < 0.05) of SPSS 16.0 software (SPSS Inc.). Columns labeled with different letters mean significantly different.

We first examined the effects of different bactericidal antibiotics carbenicillin (Carb), cefotaxime (Cef) and timentin (Tim) on bud induction. The results showed that *Agrobacterium* growth was effectively inhibited by all antibiotics at 50, 100, and 150 mg/L gradients. However, the shoot induction rates of explants cultured on media with different bactericidal antibiotics were largely different. This statistic showed that explants cultured on medium containing 150 mg/L Cef reached the highest shoot induction rate (approximately 60.0%), while explants cultured on medium containing 100 mg/L Tim reached the lowest shoot induction frequency (approximately 10.0%) (Figure 4a; Table S2). Therefore, 150 mg/L Cef is regarded as the optimal bactericidal antibiotic to eliminate *Agrobacterium* overgrowth during shoot induction in *C*. *pumila*.

To greatly increase the transformation efficiency and improve the stability of the transformation system in *C. pumila*, we systematically re-assessed the parameters affecting *Agrobacterium* inoculation and positive adventitious shoot selection. By investigating the effect of acetosyringone (AS) concentration on *C. pumila* transformation efficiency, we found that low concentration of AS (50 μM) had a positive effect on promoting genetic transformation in *C. pumila* (Figure 4b; Table S3). However, an increase of AS concentration to 100 μM led to a much lower transformation frequency, and further increase to 200 μM resulted in the failure to obtain GUS-positive transformants (Figure 4b).

In the previous report, *Agrobacterium* strain LBA4404 has been selected to inoculate *C. pumila* explants [29]. However, it is still unknown whether other *Agrobacterium* strains are also (or more) efficient for *C. pumila* genetic transformation. Here, we compared three kinds of strains – EHA105, GV3101, and LBA4404 – for their transformation efficiency in *C. pumila*. Our results showed positive transgenic plantlets can be produced on explants infected by all three strains. Most importantly, approximately 50% of explants infected by LBA4404 gave rise to GUS-positive transgenic plantlets. The lowest transformation efficiency with 15% explants producing GUS-positive plantlets was obtained when explants were infected by EHA105. The transformation efficiency of explants infected by GV3101 was higher than 20% (Figure 4c; Table S4). These results suggest that *C. pumila* explants are susceptible to a wide spectrum of *Agrobacterium* strains for high-efficient transformation, facilitating its broad application.

Comparative analyses of different combined incubation conditions including *Agrobacterium* concentration and infection time showed that the highest transformation frequency was achieved when leaf explants were inoculated in cultures with OD600 values between 0.4 and 0.6 for 20 min (Figure 4d; Table S5). Under these OD600 values, both shorter (10 min) and longer (30 min) infection time led to lower transformation frequency or fail in transformation. Once the OD600 value of *Agrobacterium* was increased to 0.8, very few or no transformants were achieved regardless of the duration of inoculation (Figure 4d; Table S5). Therefore, the improved incubation condition is immersing explants in *Agrobacterium* cultures with OD600 from 0.4 to 0.6 for 20 min.

We also re-assessed the optimal co-culture condition after *Agrobacterium* infection in *C. pumila* by considering the combined effect of co-culturing temperature and duration. As shown in Figure 4d, explants co-cultured at 26 °C for three days reached the highest transformation efficiency with approximately 40%, while two days of co-culture at 26 °C greatly reduced the transformation frequency to 20%. Similarly, three days of co-culture obtained a higher transformation rate than two days of co-culture when the temperature was reduced to 24 °C (Figure 4e; Table S6). It is interesting that the condition of being co-cultured for two days achieved a higher transformation rate than for three days at low temperature (22 °C) (Figure 4e; Table S6). Taken together, the recommended condition is co-culturing the immersed explants at 26 °C for three days.

**Figure 5:**
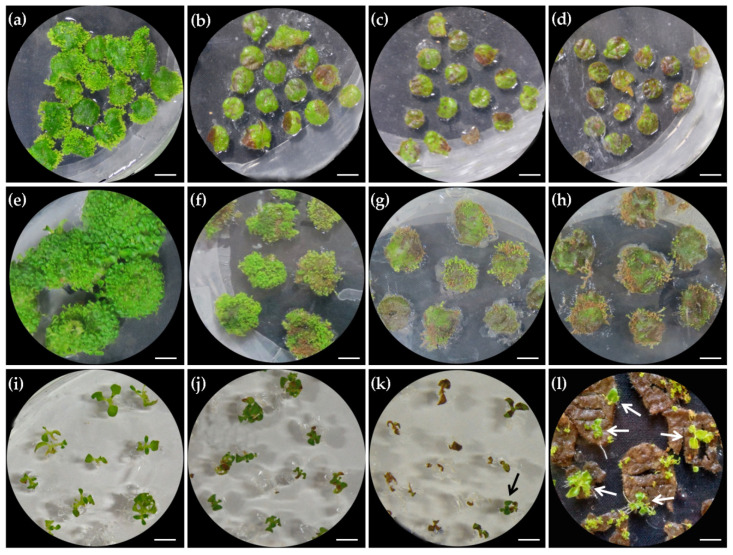
Optimization of hygromycin (Hyg) concentration for selecting positive transgenic lines at different stages. (**a**–**d**) The effect of Hyg concentration on bud induction: (**a**) 0 mg/L Hyg; (**b**) 10 mg/L Hyg; (**c**) 15 mg/L Hyg; (**d**) 20 mg/L Hyg. (**e**–**h**) The effect of Hyg concentration on adventitious bud growth. (**e**) 0 mg/L Hyg; (**f**) 5 mg/L Hyg; (**g**) 10 mg/L Hyg; (**h**) 15 mg/L Hyg. (**i**–**l**) The effect of Hyg concentration on adventitious bud elongation. (**i**) 0 mg/L Hyg; (**j**) 5 mg/L Hyg; (**k**) 10 mg/L Hyg; (**l**) The growth of negative and positive (marked by arrows) transgenic shoots, showing the rhizogenesis of positive shoots and their normal elongation on explants. Scale bars: 0.5 cm.

Hygromycin (Hyg; 20 mg/L) was previously used for effective selection of positive transgenic plants [27], but the excessive supplement of Hyg also severely represses the induction and growth of transgenic buds. To reduce the negative effect of selection pressure on positive transgenic bud induction and shoot growth, we precisely re-assessed the optimal dosage of Hyg for different stages. At bud induction stage, we found that 10 mg/L Hyg was enough to prevent untransformed explants from differentiation (Figure 5a,b). With the increase of Hyg dosage to 15 and 20 mg/L, the death rate of explants became higher (Figure 5b–d). At the post-regeneration stage, 5 mg/L Hyg showed an obvious negative effect on the growth of adventitious buds kept on explants, although almost all buds became larger (Figure 5e,f). When Hyg was increased to 10 mg/L, the negative transgenic buds were heavily inhibited in growth, and some even killed (Figure 5g). When the concentration of Hyg was further increased to 15 mg/L, nearly all buds were killed (Figure 5h). For the elongation of shoots cut from explants, 5 mg/L Hyg failed to kill any shoots but obviously injured their organs, while 10 mg/L Hyg could effectively kill all negative transgenic shoots (Figure 5i–k). In addition, we found it would be better to excise positive transgenic shoots from explants after rhizogenesis (Figure 5k,l). When 10 mg/L Hyg was used to select the positive shoots kept on explants, further elongation of untransformed shoots was completely inhibited and failed to root, while further growth and successful rhizogenesis of positive transgenic shoots were nearly unaffected (Figure 5l). Therefore, 10 mg/L, rather than 20 mg/L of Hyg [29], is enough for screening positive transgenic lines throughout the whole transgenic process.

Moreover, another widely used selection reagent called phosphinothricin (PPT) was assessed for its ability in selecting positive transgenic plantlets in *C. pumila*. Our results showed that 2 mg/L PPT was enough to completely kill wild type explants (Figure S2a,f), indicating that *C. pumila* is sensitive to PPT. We then determined the optimal concentration of PPT for selecting transformants by culturing inoculated leaf explants on selection medium containing 0, 2, 5 or 10 mg/L of PPT. We found that 2 mg/L PPT had nearly no effect on bud induction and growth (Figure S2b,c). When the PPT concentration was increased to 5 or 10 mg/L, the induction rate of transformed explants was severely decreased and bud growth was restrained (Figure S2d,e). At shoot elongation stage, 2 mg/L PPT was enough to kill wild type and negative transgenic lines, while it had little negative effect on the development of positive transgenic ones (Figure S2g,h). Further increases in PPT concentration could obviously inhibit the growth of positive transgenic shoots (Figure S2i,j). In conclusion, we suggest 5 mg/L of PPT as the optimal dosage for resistant shoot induction, while 2 mg/L for positive transgenic shoot elongation.

**Figure 6:**
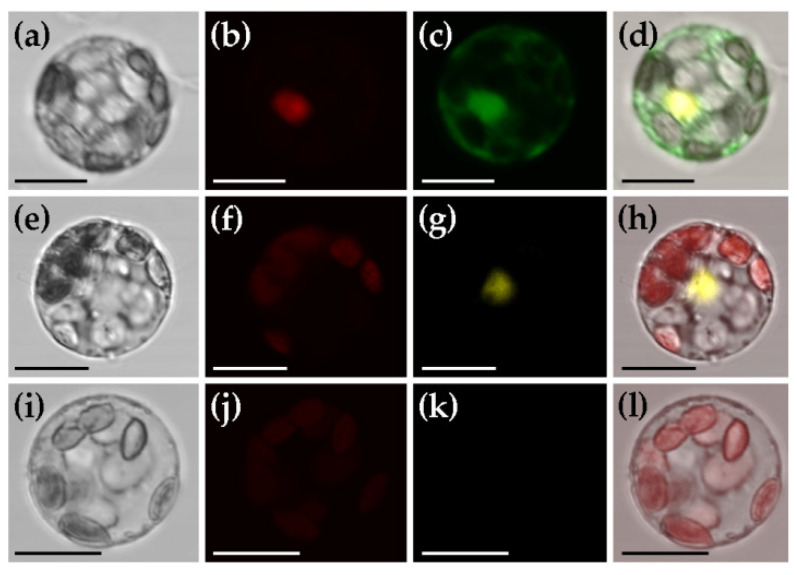
Subcellular protein localization and protein-protein interaction analyses using the transient gene expression system of C. pumila mesophyll protoplasts. (**a**–**d**) Subcellular protein localization. (**a**) Bright field image of mesophyll protoplast; (**b**) m-Cherry fluorescence detected in nucleus; (**c**) GFP fluorescence localized in both nucleus and cytoplasm; (**d**) Merged image. (**e**–**l**) Protein-protein interaction analyses between TDR1 and bHLH142. (**e**–**h**) Experimental group co-transfected with TDR1-YFP^N^ and bHLH142-YFP^C^; (**i–l**) Control group co-transfected with TDR1-YFP^N^ and YFP^C^. (**e**,**i**) Bright field image of mesophyll protoplast; (**f**,**j**) Chloroplast autofluorescence image; (**g**,**k**) YFP fluorescence image indicating the interaction between TDR1 and bHLH142; (**h**,**l**) Merged image. Scale bars: 20 µm.

To rapidly determine the basic features of a new protein of interest, such as subcellular localization and potential protein–protein interactions, it is of great convenience to test these in a transient gene expression system before stable transformation. For this purpose, we checked if these can be realized in mesophyll protoplasts of *C*. *pumila*. Excitingly, we detected the subcellular protein localization of GFP in both nucleus and cytoplasm, while H2B-mCherry in nucleus exclusively (Figure 6a–d). To check the availability of *C*. *pumila* transient gene expression system in detecting protein–protein interactions by bimolecular fluorescence complementation assay, we co-transformed a pair of interacted proteins TDR1 and bHLH142 [46] fused to YFP^N^ and YFP^C^, respectively (plasmids TDR1-YFP^N^ and bHLH142-YFP^C^). As expected, we observed obvious YFP fluorescence in the nucleus of protoplasts co-transformed with TDR1-YFP^N^ and bHLH142-YFP^C^ (Figure 6e–h), while no fluorescence in the cells transformed with TDR1- YFP^N^ and YFP^C^ (Figure 6i–l). These results indicate that the transient gene expression system of *C*. *pumila* mesophyll protoplasts is competent, at least, in analyzing the subcellular protein localization and potential protein–protein interactions.

### 2.3. Functional Analyses of CpCYC and its Promoter

**Figure 7:**
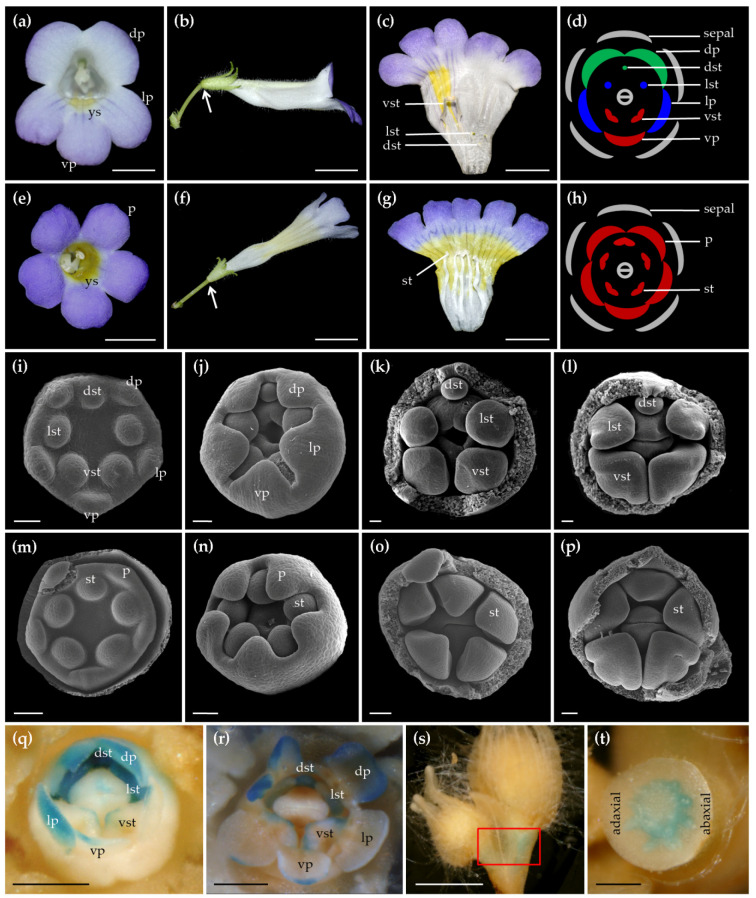
Phenotypic analyses of transgenic plants carrying *RNAi:CpCYC*. (**a**) The front view of a wild type zygomorphic flower showing three different types of petals along the dorsoventral axis and characteristic yellow spots in the ventral corolla tube. (**b**) The lateral view of the wild type flower. (**c**) A dissected flower with both dorsal and lateral stamens aborted and only two fertile ventral stamens. (**d**) Floral diagram of wild type zygomorphic flowers. (**e**) The front view of an actinomorphic transgenic flower with five identical petals and expansion of yellow spots all over the internal surface of corolla tube. (**f**) The lateral view of the transgenic flower. (**g**) The dissected transgenic flower showing five fertile stamens and expanded yellow spots. (**h**) Floral diagram of actinomorphic transgenic flowers. White arrows in panels (**b**,**f**) show the different orientation of wild type and transgenic mutant flowers. (**i**–**l**) Early development of wild type flowers showing the retardation of dorsal and lateral stamen primordia relative to the ventral ones. (**m**–**p**) Early development of *RNAi:CpCYC* plants showing synchronous development of all stamens giving rise to five fertile stamens. (**q**,**r**) GUS signals were detected mainly in dorsal petals as well as dorsal and lateral staminodes. (**s**) GUS signals were detected mainly in the pedicel (red rectangle) with a dorsoventrally asymmetric pattern. (**t**) The transverse image of the pedicel in (**l**). dp/lp/vp, dorsal/lateral/ventral petals; dst/lst/vst, dorsal/lateral/ventral stamens; p, petals; st, stamens; ys, yellow spots. Scale bars: 0.5 cm in panels (**a**–**c**,**e**–**g**,**q**–**s**); 50 μm in panels (**i**–**p**); 1 mm in panel (**t**).

To investigate the function of *CYC*-like TCP gene in *C. pumila*, *CpCYC*, we knocked- down it with RNAi technology using the transformation system optimized in this study. We obtained 64 T1 positive transgenic lines that were confirmed by PCR. Out of them, 18 independent lines showed evidently different flower phenotypes from wild type ones. As described before [29], wild type zygomorphic flowers produced three different types of petals (two dorsal, two lateral and one ventral petals) and only two fertile stamens in the ventral region. By contrast, transgenic flowers were actinomorphic with five petals identical in both size and shape, all stamens fertile and the corolla tube ventralized (Figure 7a–h), similar to the naturally occurred *peloria* flowers [47]. In addition, the yellow spot characteristic of the ventral corolla tube of wild type flowers has extended to all over the corolla tube in transgenic flowers (Figure 7a,c,e,g). Furthermore, the orientation of *RNAi:CpCYC* transgenic flowers became upright, parallel to the pedicel, different from the horizontal orientation in wild type flowers (Figure 7b,f). Quantitative real-time PCR (qRT-PCR) results showed that *CpCYC* was specifically expressed in dorsal and lateral staminodes, as well as dorsal petals in wild type flowers (Figure S3). However, *CpCYC* expression was nearly undetectable in corresponding regions of actinomorphic transgenic flowers (Figure S3). In other positive transgenic lines, flowers showed varying degrees of loss of floral zygomorphy even within a single plant (Figure S4). For example, some flowers had actinomorphic corolla but only four fertile stamens with one dorsal stamen sterile (Figure S4a,b). Some flowers had hexamerous ventralized petals with four, five or six fertile stamens (Figure S4c–h). The formation of these partially actinomorphic flowers was very likely due to the incomplete loss of *CpCYC* function, an intrinsic feature of RNAi technology.

To check if the actinomorphic phenotype can be inherited, five representative transgenic lines were selfed to harvest T2 seeds. Scanning electron microscopy (SEM) analyses in the T2 generation showed that the development of dorsal and lateral stamen primordia became retarded relative to the ventral ones at very early stages in wild type flowers, and the retardation continued to late stages of flower development (Figure 7i–l). On the contrary, the growth of all stamens in *RNAi:CpCYC* plants was nearly synchronous from early to late stages of floral development, giving rise to five fertile stamens (Figure 7m–p). In addition, we recorded the segregation ratio by calculating Hyg-resistant and -sensitive T2 seedlings of five representative lines germinated on selecting medium (1/2 MS medium supplemental with 0.02 mg/L NAA and 10 mg/L Hyg). The segregation ratio was close to 3:1, in accordance with Mendelian inheritance (Table S7), indicating that these transgenic lines were resulted from a single-copy insertion of T-DNA. Moreover, while Hyg-resistant seedlings grew healthily with well-developed root systems, Hyg-sensitive ones were malformed, similar to wild type seedlings germinated on the selection medium (Figure S4i–k). These results indicated that the actinomorphic flowers produced by transgenic lines were caused by the loss of *CpCYC* function.

To investigate the in situ *CpCYC* expression pattern, we transformed a *CpCYC* promoter:GUS reporter vector into *C*. *pumila*. The GUS signals in positive transgenic lines were mainly observed in developing dorsal petals, dorsal, and lateral staminodes (Figure 7q,r) [47], demonstrating the role of *CpCYC* in controlling the development of these floral organs. This GUS staining patterns are consistent with the expression domains of *CpCYC* tested by qRT-PCR. In addition, we detected an asymmetric GUS staining pattern, for the first time, in the pedicel with obvious stronger signal in the abaxial side than that in the adaxial side (Figure 7s,t). This GUS staining pattern reflecting the native *CpCYC* expression pattern would well explain the asymmetric growth of the pedicel (white arrow in Figure 7b); thereafter, the horizontal orientation of flowers in *C. pumila*, which become upright in RNAi transgenic lines due to the loss of function of *CpCYC* (Figure 7f). These results indicate that the specific *CpCYC* expression domains in dorsal petals, dorsal and lateral staminodes, as well as pedicels are controlled by *cis*-regulatory elements located within its promoter.

## 3. Discussion

### 3.1. A Stable and High-Efficiency Agrobacterium-Mediated Transformation System in C. pumila

Enough healthy explants are raw materials for genetic transformation. MS medium rich in macro- and micro-elements provides necessary nutrients for seed germination and plant growth [48]. Sugars are necessary to maintain the normal growth of plant cells, tissues, organs and whole plants [49]. As the most widely used tissue culture medium, MS with 3% sucrose is frequently used as recommended [50]. However, it has been reported that reduced strengths of MS salts could enhance seed germination in some species [51,52], and high concentration of exogenous sugars usually delays germination and prevents seedlings from expanding cotyledons and developing leaves [53,54]. In this study, we find that medium with low MS strength (1/2 MS) and low sucrose (1%) can speed up seed germination in *C. pumila*. Meanwhile, 1/2 MS medium with higher concentration of sucrose (2%) is required to produce healthy seedlings with stretched leaves and internodes during elongation stage.

The effect of MS salts and sucrose might be more prominent for in vitro plant regeneration systems, as it is not a fully autotrophic process and is dependent on external nutrients [55]. Here, we show that reduced MS salts and sucrose have an obvious effect to proper reducing the number of adventitious buds per explant in *C. pumila*. This finding provides a simple way to resolve the problem of excessive regeneration of clustered adventitious shoots in *C. pumila*, in which all individual buds grow slowly and some become vitrified [29]. Sugars added in the culture medium are not only as carbon sources, but also as an important osmotic agent [49]. The requirements of diverse sugar dosages for different taxa, even for different developmental stages within one species, might be due to the balance between nutrient requirement and osmotic equilibrium.

The antibiotics Carb, Cef and Tim widely used to eliminate *Agrobacterium* after co-cultivation have been reported to have an obvious influence on plant regeneration [45]. In this study, we show that both Carb and Tim exhibit a greater inhibitory effect than Cef on shoot induction from leaf explants in *C. pumila*. According to Holford and Newbury [56], one of the breakdown products of Carb is phenylacetic acid, a naturally occurring auxin which is a crucial plant growth regulator affecting regeneration. Tim also acts like a phytohormone and has a positive effect on increasing shoot regeneration efficiency [57,58]. The significant differences of plant growth regulators in both kinds and their dosages have been widely reported for shoot regeneration among species. The significant effect on shoot induction caused by Carb, Cef and Tim might be attributed to the breakdown products that disturb the endogenous auxin/cytokinin balance in *C. pumila*.

High-efficiency T-DNA delivery from *Agrobacterium* into plant cells is a prerequisite for a successful transgenic system. Acetosyringone (AS) is the most frequently used phenolic to induce the expression of *Agrobacterium vir* gene directing genetic transformation of T-DNA from *Agrobacterium* to plant cells [59,60,61]. However, the required AS dosage is quite different even among closely related groups [62]. In the initial study reporting the development of *C*. *pumila* transformation system, 150 mg/L (764 μM) AS was directly used without an assessment [29]. Here, we show that a lower level (50 μM) of AS has a positive effect on transformation efficiency in *C*. *pumila*, while increased levels, are detrimental. We infer that the requirement of different concentrations of exogenous AS among species might be greatly affected by inherent phenolics secreted by wounded tissues.

*Agrobacterium* incubation and co-culture with explants are complicated dynamic progresses relating to T-DNA delivery [59,63,64,65,66]. However, the optimal conditions for all these parameters are significant different among taxa. In some species, specific *Agrobacterium* strains are suitable for genetic transformation, but other strains fail to deliver T-DNA into plant cells [63,64]. Excitingly, we here confirm that any of three kinds of *Agrobacterium* strains, that is, LBA4404, GV3101, and EHA105, gives a high transformation rate in *C. pumila*. The wide availability of *Agrobacterium* strains would greatly facilitate the application of this transformation system in functional studies. The optimal incubation and co-culture condition for high transformation efficiency should reach a balance between sufficient infection and minimal explant damage. Too low a concentration of *Agrobacterium* cells and short infection time might reduce the sufficient attachment of *Agrobacterium* to explants and result in the low transfer efficiency of T-DNA from *Agrobacterium* to plant cells, while too high concentration of *Agrobacterium* cells and prolonged inoculation time would damage explants and decrease their regeneration ability. After a series of tests, we have got a balance between *Agrobacterium* cell density and infection time, that is, infecting *C*. *pumila* explants for 20 min with *Agrobacterium* cells of OD600 = 0.4 and 0.6. Both shorter inoculation with lower *Agrobacterium* cell density and prolonged infection with higher cell density give rise to low transformation efficiency.

Gene transfer from *Agrobacterium* to plant cells is thought of a temperature-sensitive process [67]. Both co-culture duration and temperature are usually variable in different transformation systems, even among different explant types of a given species [62,68]. Our results show that a synergistic effect of co-culture temperature and duration is achieved after three days of co-culture at 26 °C, generating the highest transformation efficiency. The increased transfer efficiency of longer duration (three days) at higher temperatures (24 °C and 26 °C) might be due to the fact that higher temperatures optimal for *C*. *pumila* growth lead to increased tolerance of explants to virulence from *Agrobacterium* overgrowth. However, it is surprising that short duration (two days) with low temperature (22 °C) can also bring about a high transformation rate. Thus, a balance between co-culture duration and temperature would enhance T-DNA delivery from *Agrobacterium* into plant cells.

As outlined above, we have comprehensively optimized the transformation system of *C*. *pumila* by monitoring a series of factors affecting seedling growth, plant regeneration and genetic transformation. Compared with the previous report [29], the main improvements in this study are summarized in Table 4. Typically, we have shortened the duration from wild type seeds to transgenic plants, improved shoot induction and selection conditions. We have also broadened the application of this system in both *Agrobacterium* strains (LBA4404, GV3101, and EHA105) and selection markers (Hyg and PPT). In addition, we have optimized the parameters or combination of them for improving the transformation rate and accelerating the growth of transgenic lines. Under the optimized conditions, almost all Hyg-resistant plantlets are positive, and the whole transformation duration (from *Agrobacterium* inoculation to 1–2 cm transgenic plantlets) is shortened within 2 months (Table 4).

### 3.2. Functional and Evolutionary Implications of CpCYC Silence by RNAi and Its Promoter Activity

Floral zygomorphy has been proposed as a key innovation adapting to specialized pollinators and evolved multiple times in angiosperms [27,69]. Mutant studies in *Antirrhinum*, characterized by zygomorphic flowers, have promoted the identification of major floral symmetry genes, that is, *CYC*, *DICH*, *RADIALIS* (*RAD*) and *DIVARICATA* (*DIV*) [11,12,70,71]. The genetic interactions among these genes have been characterized based on phenotypic and gene expression pattern changes benefiting from the availability of a series of genetic mutants in *Antirrhinum*. In dorsal floral regions, CYC and DICH promote *RAD* expression, whereas RAD competes with DIV for interacting with its partners and thus confines its activity to ventral floral regions [72,73]. Floral zygomorphy has evolved repeatedly from actinomorphy throughout angiosperms [74]. Currently, an increasingly growing number of researches focus on investigating the key homologous components that are responsible for flower symmetry in *Antirrhinum*, especially *CYC*-like genes, in extensive angiosperm species including non-model species with unique symmetry features or with an important phylogenetic position [75]. Expression analyses here clearly demonstrate the correlation between *CpCYC* expression pattern and the slightly shorter dorsal petals and retarded dorsal/lateral staminodes in *C*. *pumila*. Comparative studies of floral zygomorphy and *CYC*-like expression patterns carried out in diverse species suggest that asymmetric *CYC*-like gene expression is involved in the evolution of zygomorphy at least in eudicots, such as Ranunculales, Lamiales, Brassicales, Malpighiales, Dipsacales, Asterales, and Fabales, and some monocots, including Zingiberales and Commelinales [15,75,76,77,78,79]. Moreover, diverse expression domains of *CYC*-like genes, such as in the dorsal (*Antirrhinum*), lateral (*Mohavea* and *Primulina*), or ventral (*Opithandra*) regions of the flower, are clearly correlated with the development of corresponding floral organs [11,12,13,14,17].

While extensive expression data in various species suggest important roles of *CYC*-like genes in zygomorphic floral development, functional evidence is very limited. Our previous study on gene expression and phenotypic analyses on the basis of natural *peloria* indicates that *CpCYC*, a *CYC*-like gene in *C*. *pumila*, may regulate the shape of dorsal petals and repress the development of dorsal and lateral stamens in *C*. *pumila*, but this needs further functional tests [47]. In this study, we carried out functional analyses by silencing *CpCYC* using RNAi technology in *C*. *pumila*. Our results show that transgenic plants with dorsal floral organs escaping from *CpCYC* expression produce inheritable fully actinomorphic flowers with five equal petals and five fertile stamens, similar to the natural mutant *C*. *pumila* flowers [47] and the *cyc dich* double mutants in *Antirrhinum* [11]. Our results provide a direct functional evidence for the role of *CpCYC* in controlling the development of dorsal petals and dorsal/lateral staminodes in *C*. *pumila*. The function of *CpCYC* in *C*. *pumila* is consistent with the scattered functional evidence about the roles of *CYC*-like genes in floral zygomorphy reported from *Mimulus* (Phrymataceae) [80], *Lotus japonicus* and *Pisum sativum* (Legumes) [15,77], *Gerbera*, and *Senecio* (Asteraceae) [81,82] as well as *Cysticapnos* (Papaveraceae), the basic group in eudicots [83]. These functional data suggest that *CYC*-like genes have a conserved function in controlling the development of petals and staminodes within angiosperms. However, diverse phenotypic effects of *CYC*-like genes in dorsal, lateral, or ventral regions indicate that they might be independently recruited in each zygomorphic clade with the acquisition of different functions accounting for the distinct zygomorphy between lineages.

In addition, our results demonstrate a simultaneous expansion of yellow strips from the ventral to lateral and dorsal regions of the corolla tube in ventralized actinomorphic transgenic flowers upon *CpCYC* silence, reminiscent of the naturally occurred *peloria* mutants in its relative *Primulina* due to the loss-of-function of both *PhCYC1C* and *PhCYC1D* [13]. These findings indicate that *CYC*-like genes may control the asymmetric pigmentation pattern in *C*. *pumila*, as well as other members of Gesneriaceae. At an evolutionary scale, flowers are highly integrated structures in which different organs co-function, while pleiotropic genes are usually responsible for correlated variations in floral traits [84]. The pigmentation in flowers serves an important function in attracting pollinators [85]. A functional study in *Torenia fournieri* shows that *TfCYC2* controls both asymmetric corolla pigmentation and petal shape. When the expression of *TfCYC2* was down-regulated, the dorsal petals became ventralized both in shape and color pattern with violet spots. They further find that *TfCYC2* can directly bind to the regulatory regions of *TfMYB1*, an R2R3-MYB transcription factor recruited in the control of various pigmentation patterns that can negatively regulate its promoter activity [20]. In *Mimulus lewisii*, the ventral petals have two ridges covered by yellow pigmentation, similar to that in *C*. *pumila*. Decreased expression level of *RCP1*, a MYB gene, causes weakened pigmentation in two ridges, while over-expression of *RCP1* results in reduced flower size besides the complementation for *rcp1* mutant [86]. In *C*. *pumila*, the yellow pigmentation, a typical character in the ventral corolla tube of wild type flowers, is expanded to the whole corolla tube in actinomorphic *RNAi:CpCYC* flowers, hinting that *CpCYC* has a function in repressing yellow pigmentation in the dorsal corolla tube of wild type flowers. Our results further support that *CYC*-like genes might be involved in the asymmetrical pigmentation patterns of petals and corolla tubes, probably via negatively modulating MYB family genes. However, further functional analyses are required to clarify whether the pathways by which *CYC*-like genes control the floral pigmentation pattern is conserved.

In addition to the changed pigmentation pattern in corolla tube, we observe a correlative change in floral orientation from horizontal to upright accompanied with floral symmetry from zygomorphy to actinomorphy in *CpCYC* silencing flowers. In *Sinningia speciosa*, another species in Gesneriaceae, a correlated change of floral orientation and symmetry state has been reported in *SsCYC* loss-of-function transgenic lines and natural mutants [87]. Apparently, *CYC*-like genes are a kind of typical pleiotropic regulators responsible for multiple floral phenotypes relating to floral symmetry. Recent studies in animals and plants have shown that similar phenotypes are possibly evolved from multiple molecular pathways. The *yellow* gene is a famous pleiotropic gene in insects. The different *yellow* expression domains are governed by distinct *cis*-regulatory elements (CREs) [88]. The gains of *yellow* expression in wing spots of *Drosophila tristis* and *D*. *melanogaster* have evolved through the co-option and modification of distinct CREs [88]. The change of floral orientation in *Sinningia speciosa* has been reported to be due to the loss of a gibbous structure at the base of dorsal corolla tube controlled by *SsCYC* [87]. However, in *C*. *pumila*, no gibbous structure is found at the base of dorsal corolla tube. Instead, our results show for the first time that the horizontal oriented flower in *C*. *pumila* might result from an asymmetric expression of *CpCYC* (from the GUS staining data controlled by *CpCYC* promoter) at the junction of the flower and its pedicle. These data indicate that the function of *CYC*-like genes in controlling floral orientation might be recruited by diverse molecular pathways via distinct *cis*-regulatory elements. It has been reported that zygomorphy has independently evolved at least 130 times [74]. The great diversity of floral zygomorphy provides an excellent opportunity to test whether they evolve using similar or different mechanisms. To address these questions, a major goal of future research will be to clarify how the *CYC*-like genes’ activities in particular floral organs are regulated by upstream *cis*-elements and *trans*-factors and how they interact with other transcription factors.

### 3.3. Biological Advantages Make Chirita Pumila as a Potential Model Plant

One of the main topics of evo-devo biologists is to understand how the function and regulatory pathways of important regulatory genes have evolved to generate the conservation and diversification of key morphological novelties in diverse species. As a first step to achieving this goal, it is urgently required to conduct functional and regulatory studies from a range of species representing crucial phylogenetic clades. To date, a series of model systems have been developed to address these questions. For example, pioneering researches in both *Antirrhinum* and *Arabidopsis*, two classical model plants respectively belonging to Asteridae and Rosidae, have played pivotal roles in elucidating the molecular pathways underlying the determination of floral organ identity and have directly led to the proposal of the famous ABC model [4,5]. *Antirrhinum* is also a classical model to uncover the molecular mechanisms underlying the origin of floral zygomorphy [11,12,71,72]. In addition, *Petunia* has been widely used as a model in flower development, retroelement activity and male sterility based on its diversity in mutants [38], and *Aquilegia* has been developed as a model for speciation genetics due to its basal phylogenetic position [24]. *Mimulus* has long been recognized as a classic ecological and evolutionary model system [35]. *Lotus japonicus* and *Medicago* are representatives in addressing some specific events of legumes, such as nodules [32,33,34]. *Populus* and rice have been respectively used as models in trees and grasses [28,41]. *Chirita pumila*, as a representative of Gesneriaceae, has many advantages common to model plants as aforementioned. *Chirita pumila* is an ideal species to be developed as a model system. The easy availability of both stable and transient transformation systems in *C*. *pumila* meets the demand for large-scale functional studies in its native system. The biological features together with the efficient stable and transient transformation systems would greatly facilitate genetic and functional analyses in *C*. *pumila* addressing a broad range of evo-devo questions, especially in the field of floral evolution.

Although the classical ABC model was initially proposed based on homeotic mutants in *Arabidopsis* and *Antirrhinum*, little progress has been achieved in *Antirrhinum* in the past two decades because of lack of transformation system in this model [7,89,90]. Furthermore, in contrast to the conserved B- and C-function in floral organ identity, the A-function floral identity genes are limited to *Arabidopsis* and its close relatives and they may be lacking in *Antirrhinum* [7,90]. To understand these differences between species, comparative gene function analyses in a broad range of lineages are critically required. *Chirita pumila* is a member of Gesneriaceae, an early deriving lineage from Lamiales (Figure 1; APG IV). The efficient genetic transformation system would make *C*. *pumila* become a new model of Lamiales to enrich the content of the classical ABC model, exampled by a recent finding of one of A-class genes involved in controlling floral determinacy in *C*. *pumila*, and to clarify the molecular relationship between floral zygomorphy and floral organ identity. Interestingly, *C*. *pumila*, characterized by large zygomorphic flowers adapting to specific pollinators for cross-pollination, always finishes self-fertilization before anthesis [29]. This feature also makes *C*. *pumila* as a perfect model to uncover the morphological and genetic mechanisms of the change in mating system. The specific DNA content, the major force in genetic diversity, is embedded in chromosome. Differences in chromosome number, shape, and size are all subjected to evolution. The great different karyotype of *C*. *pumila* (2 *n* = 8) from its close relatives (2 *n* = 18) will provides a wonderful platform for researchers to address the mechanism of chromosome evolution as suggested before [29].

Furthermore, in contrast to the subfamily Gesnerioideae with two equal cotyledons in seedlings, the plants of the subfamily Cyrtandroideae, including *C*. *pumila*, are characteristic of anisocotyledonous seedlings with a fully grown macrocotyledon and a strongly repressed tiny microcotyledon, which are unequally developed from an isocotylous embryo (Figure 2) [44]. Previous studies have shown that *CYC*-like genes retard the development of dorsal and dorsal/ventral stamens by locally repressing cell cycle genes [17,91]. However, it is still an open question whether anisocotyledonous development in Gesneriaceae is related to the function of *CYC*-like genes or other genes in cell proliferation and expansion. Angiosperms are traditionally classified into two major groups: dicots and monocots. Recent molecular phylogenetic data strongly support that monocots have evolved from the paraphyletic dicots (APG IV). The question about the homology of the sterile cotyledon between monocots and dicots has been a hot topic for evolutionary botany, but it remains a largely morphological speculation [92]. One theory is that one of the cotyledons was progressively reduced and ultimately lost during early monocot evolution [93]. Knowledge from functional and mechanistic studies in size difference of two cotyledons in *C*. *pumila* would shed critical light on the understanding of this complex issue. Taken together, *C*. *pumila*, as an emerging model, holds a series of great advantages for developmental and evo-devo studies of some important traits that are not found in well-established classical model systems.

## 4. Materials and Methods

### 4.1. Plant Material and Phylogenetic Analysis

*Chirita pumila* D. Don (Wang, HK01) plants were collected from Hekou County, Yunnan, China, and grown in 8 cm pots containing the mixture of vermiculite and Pindstrup substrate (Pindstrup, Ryomgaard, Denmark) (1:2) in the greenhouse of Institute of Botany, Chinese Academy of Sciences under a natural light/dark photoperiod in Beijing with relative humidity in the range of 50–70% [29]. All explants were from sterile seedlings approximately 2–3 months old cultured in the growth chamber under conditions as described [29]. All seeds were collected and stored in the 4 °C; freezer until sowing.

The chloroplast DNA regions *rbcl*, *matk* and *ndhf* of major model plants were used to construct the phylogenetic tree to identify the position of *C*. *pumila* in angiosperms using maximum marsimony methods as implemented in PAUP*4.0B10 [94]. All sequences used were retrieved from NCBI (National Center for Biotechnology Information, https://www.ncbi.nlm.nih.gov/, accessed on 16 March 2021; the accession numbers of sequences are listed in Table S8) and then aligned and adjusted manually using the software Geneious version 11.0.3 (https://www.geneious.com/, accessed on 16 March 2021). All characters were unordered and had equal weight. Gaps were treated as missing data. Starting trees were obtained via stepwise addition. Heuristic searches were carried out with 1000 replicates of random addition, one tree being held at each step during stepwise addition, with tree-bisection-reconnection branch-swapping strategy. The robustness of different clades was assessed by means of nonparametric bootstrapping [95] using 1000 replicates and a heuristic search with 1000 replicates of random sequence addition and tree-bisection-reconnection branch-swapping.

### 4.2. Determination of the Optimal Media for Seed Germination and Seedling Growth

To optimize the seed germination media, over 100 sterilized seeds were germinated on six kinds of media SGM-I, SGM-II, SGM-III, SGM-IV, SGM-V, and SGM-VI with different strengths of MS salts and different concentrations of sucrose, respectively (Table 2). The germination time of nearly all seeds was recorded for each medium. The medium on which seed geminated most quickly is taken as the most optimal for seed germination. Four weeks later, the growth states of seedlings were assessed. Then, seedlings germinated from the optimal medium were dividedly sub-cultured onto SGM-I, SGM-II, SGM-III, SGM-IV, SGM-V, and SGM-VI. Three weeks later, the seedling growth states were assessed once again.

To measure whether NAA affects seed germination, over 100 sterilized seeds were germinated on the optimal medium additionally supplemented with 0, 0.02, 0.05 or 0.1 mg/L NAA, respectively. The germination time of nearly all seeds was recorded for each medium. Four weeks later, the growth states of seedlings were assessed.

### 4.3. Optimization of Tissue Culture Conditions

We first detected the effect of placement way of explants on bud induction. Leaf explants were placed on TCM-III [29] with adaxial or abaxial leaf surfaces upward. Two weeks later, the induction rate of adventitious buds was recorded. To assess the effect of phytohormone on adventitious bud growth, adventitious shoots maintained on explants were transferred to fresh TCM-III medium with or without 0.5 mg/L 6-BA and 0.1 mg/L NAA. The growth state of adventitious shoots was assessed after another two weeks.

To determine the optimal shoot induction medium, leaf explants were, respectively, cultured on TCM-I, TCM-II, TCM-III, TCM-IV, TCM-V and TCM-VI media at 26 °C. Once adventitious buds were induced (about two weeks later), the explants together with adventitious buds were, respectively, sub-cultured to corresponding fresh media without 6-BA and NAA. The effect of different media on adventitious shoot induction and shoot growth state were recorded one month later. To determine the optimal shoot elongation and rooting medium, the adventitious shoots from the optimal shoot induction medium were dissected from the explants and independently transferred onto fresh SGM media (SGM-I, SGM-II, SGM-III, SGM-IV, SGM-V and SGM-VI). The rooting and growth state of adventitious shoots were recorded three weeks later.

### 4.4. Improvement of Acetosyringone Concentration and Antibiotics Usage for C. pumila Transformation System

To determine the optimal AS concentration, a single colony of *Agrobacterium* EHA105 harboring the binary vector pCAMBIA1301 was cultured in 5 mL YEB selection medium (containing 50 mg/L kanamycin and 50 mg/L rifampicin) with shaking (180 rpm) at 28 °C overnight. The next day, 2 mL of overnight cultures was inoculated into 50 mL fresh YEB selection medium supplemented with 0, 50, 100, or 200 μM AS and grown to OD600 ≈ 0.6. The cells were harvested by centrifuging at 5000 rpm for 5 min, rinsed with immersing medium (IMM) containing different concentrations of AS corresponding to the YEB selection medium, and then re-suspended in isometric IMM liquid. Fresh explants were inoculated in the harvested cells at room temperature for 20 min, and then incubated on TCM-III additionally supplemented with different concentrations of AS at 26 °C in the dark for two days. Then, the explants were transferred to the shoot induction medium SIM-I (containing 20 mg/L Hyg). Four weeks later, transformation frequency (the ratio of positive explants confirmed by PCR) was recorded. PCR was conducted using primers spanning the 35S promoter and the *GUS* gene as illustrated before [29].

To optimize the Hyg dosage for screening positive transgenic lines (LBA4404 strain harboring the pCAMBIA1301 vector was used) at different stages, explants were immersed in *Agrobacterium* suspension liquid (OD600 = 0.6) containing 150 μM AS at room temperature for 20 min; co-cultured on TCM-III supplemented with 150 μM AS at 26 °C in the dark for two days; and then transferred onto SIM-I containing 0, 10, 15, or 20 mg/L of Hyg, respectively (Table 2). Two months later, the induction states were recorded, and the buds (together with the leaf discs) induced from medium without Hyg were independently transferred to fresh selection medium SSM-I containing 0, 5, 10, and 15 mg/L Hyg, respectively (Table 2). Four more weeks later, the growth states of seedlings were recorded. Seedlings grown on medium without Hyg were cut off from the discs and once more independently sub-cultured to fresh SSM-I containing 0, 5, 10, and 15 mg/L Hyg, respectively (Table 2). Three weeks later, the growth states of seedlings were recorded again.

In addition, to extend the application of *C. pumila* transformation system, we assessed the effect of PPT, as a selection marker, on screening positive transgenic lines. LBA4404 strain containing the pCAMBIA3301vector with PPT resistance gene *bar* was used. Explants were immersed with *Agrobacterium* and co-cultured as mentioned for Hyg. Then, the co-cultured explants were first transferred onto SIM-III with 0, 2, 5, and 10 mg/L PPT, respectively (Table 2). Four weeks later, the induction rate of explants was recorded, and the shoots induced from medium without PPT were cut off from the explants and independently transformed to SSM-II medium with 0, 2, 5, and 10 mg/L PPT, respectively. Two more weeks later, PPT-resistant lines were recorded and confirmed by PCR using primers spanning the 35S promoter and the *GUS* gene [29]. As a control, the effect of PPT on wild type explants and seedlings was also detected.

To find the most efficient bactericidal antibiotics for eliminating *Agrobacterium* overgrowth, explants were immersed with *Agrobacterium* and co-cultured as mentioned for Hyg. Then, co-cultured explants were transferred onto the SIM-II containing 50, 100 or 150 mg/L of Carb, Cef or Tim, respectively (Table 2). Four weeks later, the induction rate of leaf discs was recorded.

### 4.5. Determination of Agrobacterium Strains, Explants Inoculation and Co-Culture Conditions for High-Efficiency C. pumila Transformation System

The effect of *Agrobacterium* strains on transformation efficiency was assessed using *A*. *tumefaciens* strains LBA4404, GV3101, and EHA105 carrying the binary vector pCAMBIA1301. Fresh explants were inoculated in harvested cells at room temperature for 20 min, and then incubated on CCM at 26 °C in dark for three days. Then, the explants were transferred to the shoot induction medium SIM-III. Four weeks later, the transformation rates (the ratio of positive explants confirmed by PCR as mentioned above) were recorded.

The effect of immersing and co-culture conditions on transformation efficiency of *C. pumila* explants was assessed using EHA105 strain harboring the binary vector pCAMBIA1301. To assess the effect of immersing conditions, explants were immersed in *Agrobacterium* of different OD600 values (0.4, 0.6, or 0.8) for 10, 20, or 30 min, and then co-cultured at 26 °C in the dark for two days. To determine an optimal co-culture condition, fresh explants were inoculated in *Agrobacterium* with a OD600 value of 0.4–0.6 at room temperature for 20 min, and then separately co-cultured at 22, 24 or 26 °C for two or three days in the dark. For both experiments, the co-culture explants were selected on SIM-III media containing 10 mg/L Hyg and 150 mg/L Cef. Four weeks later, the transformation efficiency (the ratio of positive explants confirmed by PCR as mentioned above) was recorded.

### 4.6. Statistical Analyses

To assess the effects of bactericidal antibiotics (types and their dosage) on bud induction, we compared their induction frequency (ratio of explants with regenerated buds to total explants). To assess the effects of some factors (AS concentration, *Agrobacterium* strains, immersing and co-culture conditions) on the transformation efficiency in *C*. *pumila*, we calculated their positive transformation rates (ratio of explants with Hyg-resistant buds confirmed by PCR to total explants). Both the induction efficiency and transformation rate are from three independent replicates with each containing 30–45 explants. Data presented are mean ± SD from the replicates. The difference level of significance among groups was analyzed using a Fisher’s LSD test (*p* < 0.05) under One-Way ANOVA module of SPSS 16.0 software (SPSS Inc., Chicago, IL, USA).

### 4.7. Protoplast Isolation and Transient Gene Expression Assay

*Chirita pumila* leaves were used for protoplast isolation as described in Arabidopsis [96] with some modifications. Briefly, protoplasts were collected by diluting with W5 solution (2 mM MES pH 5.7, 154 mM NaCl, 125 mM CaCl_2_, 5 mM KCl) to a final concentration of  10^6^ cells/mL. Then, the protoplasts were resuspended in MMG solution (4 mM MES pH 5.7, 0.4 M mannitol, 15 mM MgCl_2_). For transfection, totally 10^5^ protoplasts in 100 μL were mixed with 10 μg plasmid and 110 μL PEG solution (40% PEG4000, 0.2 M mannitol, 0.2 M CaCl_2_). After incubation, 440 μL of W5 solution was added. Protoplasts were harvested and resuspended in WI solution (4 mM MES pH 5.7, 0.5 M mannitol, 20 mM KCl) overnight. HBT-GFP (kindly gifted by Professor Chun-Ming Liu) and pBI221-H2B-mCherry (kindly gifted by Professor Kang Chong) plasmids were used for subcellular localization analyses, while TDR1-YFP^N^, bHLH142-YFP^C^ and YFP^C^ plasmids (kindly gifted by Professor Yao-Guang Liu) were used for protein-protein interaction analyses.

### 4.8. Functional Studies of CpCYC and its Promoter Using C. pumila Transformation System

In our previous study, we isolated a single *CYC*-like gene in *C*. *pumia* – *CpCYC*– belonging to the GCYC2 Clade [47]. The sequences of the *CpCYC* gene have been deposited in the GenBank database under accession numbers MT023082 (for the full-length promoter) and MT023083 (for the full-length coding region). Function analyses of *CpCYC* gene were carried out using RNAi technology. The RNAi:CpCYC plasmid was constructed as follows: the fragment of *CpCYC* gene was amplified using gene-specific primers (forward: 5′-GACTAGTGGCGCGCCGAGTTCTTGCTGCATCACCACC-3′, *Spe* I and *Asc* I recognition sites are, respectively, single and double underlined; reverse: 5′-AGGGATCCATTTAAATCTGTGCCGATCTTTCTTCACCG-3′, *Bam*H I and *Swa* I recognition sites are respectively single and double underlined). The PCR product was digested by *Asc* I and *Swa* I, and then inserted into the corresponding sites of pFGC1008 to obtain the RNAi:CpCYC sense plasmid that was confirmed by DNA sequencing. Then, the same PCR product was digested by *Spe* I and *Bam*H I and inserted into the corresponding sites of the RNAi:CpCYC sense plasmid (Figure S5). The resultant RNAi:CpCYC plasmid was confirmed by DNA sequencing before being introduced into *Agrobacterium* LBA4404 by heat shock. *CpCYC* promoter:GUS vector was constructed using the full-length promoter as described [47].

Transformation was carried out using the optimized conditions mentioned above. Candidate positive transgenic plantlets (Hyg-resistant) of 1–2 cm in height were confirmed by PCR (forward: 5′-GCGCGTGACAAAAACCACC-3′; reverse: 5′-GAACCCTGT GGTTGGCATGCAC-3′) and sequencing. Positive transgenic plants were planted to the greenhouse. To analyze the *CpCYC* expression pattern in transgenic plants, RNA from transgenic and wild type flowers was respectively extracted from dorsal/lateral/ventral petals and dorsal/lateral/ventral stamens. RNA extraction and complementary DNA synthesis were conducted as described [19]. Gene-specific primers (forward: 5′-CTCGCG CCTCTACTTCTGTCGTG-3′; reverse: 5′-CGTGTTGGCCATGGTAGAATTAGGG-3′) were used to amplify *CpCYC* gene. As a reference gene, *CpACTIN* gene was also amplified using gene-specific primers (forward: 5′-AGTTATCACCATTGCCGCCGAGAGG-3′; reverse: 5′-GCAATGCCAGGGAACATAGTCGACC-3′). The specificity of all primers was confirmed by sequencing their PCR products. qRT-PCR and data analyses were performed as described previously [19]. The phenotype of *CpCYC* down-regulated lines was recorded.

To validate the hereditability of mutant phenotype observed in T1 plants, T2 seeds were disinfected and selected using 10 mg/L Hyg. The ratio of the number of Hyg-resistant and -sensitive seedlings was calculated. Floral phenotypes of Hyg-resistant T2 plants were also recorded. Wild type seeds were sowed simultaneously as a control. For scanning electron microscopy, floral buds of different developmental stages from both wild type plants and T2 generation of RNAi plants were collected and imaged using a Hitachi S-4800 scanning electron microscope as described [47]. GUS staining was conducted as described [47].

## 5. Conclusions

We systematically optimized the conditions for seed germination, tissue culture and genetic transformation in *C. pumila*. In addition, we detected the availability of the transient gene expression system of *C*. *pumila* mesophyll protoplasts in subcellular protein localization and protein-protein interaction analyses. Moreover, we carried out functional studies of *CpCYC* using the improved transformation system. Our results reveal the roles of *CpCYC* in regulating the development of dorsal petals and dorsal/lateral staminodes, repressing the formation of yellow strips in the dorsal corolla tube and determining the asymmetric growth of the pedicle. We also reveal the role of *CpCYC* promoter in controlling spatial *CpCYC* expression patterns in the pedicle, dorsal petals, and dorsal/lateral staminodes. Now that the transformation efficiency and stability of the genetic transformation system have been greatly improved in this study, *C. pumila* can be used for large-scale function investigations of related genes and regulatory elements. Together with the development of transient gene expression system and the completion of genome sequencing project, *C. pumila* would undoubtedly become an ideal model plant for studies in the field of evo-devo, such as the evolution of the molecular networks in floral organ identity, floral zygomorphy, and anisocotyledonous in Gesneriaceae.

## Figures and Tables

**Table 1:** Comparison among representative model plants.

Species	Generation Time (Month)	Genome and Karyotype	Plant Height (cm)	Seeds per Plant	Stable Transformation	Transient Transformation	References
*Antirrhinum majus*	perennial	510 Mb, 2 *n* = 16	20–90	hundreds	difficult	no	[22,23]
*Aquilegia*	3–12	400 Mb, 2 *n* = 14	20–25	hundreds	no	available	[24]
*Arabidopsis thaliala*	1–2	125 Mb, 2 *n* = 10	30	thousands	simple	available	[28]
*Chirita pumila*	4–5	798.7 Mb, 2 *n* = 8	6–46	thousands	simple	available	[29], this study
*Glycine max*	5–6	1100 Mb, 2 *n* = 20	30–90	hundreds	difficult	available	[25,26]
*Helianthus annuus*	3–4	3.6 Gb, 2 *n* = 34	50–500	hundreds	difficult	available	[30,31]
*Lotus japonicus*	3–4	450 Mb, 2 *n* = 12	30	thousands	simple	available	[32]
*Medicago truncatula*	2.5–3.5	520 Mb, 2 *n* = 16	50–100	thousands	simple	available	[33,34]
*Mimulus*	2.5–3	500 Mb, 2 *n* = 28	20–30	thousands	simple	available	[35]
*Nicotiana benthamiana*	2–3	3.1 Gb, 2 *n* = 38	40–200	hundreds	simple	available	[36,37]
*Oryza sativa*	3–6	466 Mb, 2 *n* = 24	50–150	hundreds	simple	available	[28]
*Petunia hybrida*	3–4	1.3 Gb, 2 *n* = 28	30–60	thousands	difficult	available	[38,39]
*Populus trichocarpa*	perennial	480 Mb, 2 *n* = 38	tall trees	unrecorded	difficult	no	[40]
*Solanum lycopersicum*	2–3	950 Mb, 2 *n* = 24	15–100	hundreds	simple	available	[41]

**Table 2:** Composition of media used in this study.

Media	Composition
SGM-I	MS, 1% sucrose, solid
SGM-II	MS, 2% sucrose, solid
SGM-III	MS, 3% sucrose, solid
SGM-IV	1/2 MS, 1% sucrose, solid
SGM-V	1/2 MS, 2% sucrose, solid
SGM-VI	1/2 MS, 3% sucrose, solid
TCM-I	MS, 1% sucrose, 0.5 mg/L 6-BA, 0.1 mg/L NAA, solid
TCM-II	MS, 2% sucrose, 0.5 mg/L 6-BA, 0.1 mg/L NAA, solid
TCM-III	MS, 3% sucrose, 0.5 mg/L 6-BA, 0.1 mg/L NAA, solid
TCM-IV	1/2 MS, 1% sucrose, 0.5 mg/L 6-BA, 0.1 mg/L NAA, solid
TCM-V	1/2 MS, 2% sucrose, 0.5 mg/L 6-BA, 0.1 mg/L NAA, solid
TCM-VI	1/2 MS, 3% sucrose, 0.5 mg/L 6-BA, 0.1 mg/L NAA, solid
IMM	1/2 MS, 1% sucrose, 0.5 mg/L 6-BA, 0.1 mg/L NAA, 50 μM acetosyringone, liquid
CCM	1/2 MS, 1% sucrose, 0.5 mg/L 6-BA, 0.1 mg/L NAA, 50 μM acetosyringone, solid
SIM-I	MS, 3% sucrose, 0.5 mg/L 6-BA, 0.1 mg/L NAA, hygromycin (0, 5, 10, 15 or 20 mg/L), 150 mg/L carbenicillin, solid
SIM-II	MS, 3% sucrose, 0.5 mg/L 6-BA, 0.1 mg/L NAA, 20 mg/L hygromycin, different concentrations (0, 50, 100 or 150 mg/L) of carbenicillin, cefotaxime or timentin, solid
SIM-III	1/2 MS, 1% sucrose, 0.5 mg/L 6-BA, 0.1 mg/L NAA, 10 mg/L hygromycin or different concentrations (0, 2, 5 or 10 mg/L) of phosphinothricin,150 mg/L cefotaxime, solid
SSM-I	MS, 3% sucrose, hygromycin (0, 5, 10, 15 or 20 mg/L), solid
SSM-II	1/2 MS, 2% sucrose, phosphinothricin (0, 2, 5 or 10 mg/L), solid

The pH value of all media was adjusted to 5.7–5.9. All solid media contain 4 g/L Gellan Gum.

**Table 3:** Determination of the optimal media for adventitious shoot induction and shoot elongation.

Culture Stages	MS Salt Strength	Sucrose Concentration	Number of Explants or Shoots	Description of Adventitious Shoots
Bud Induction Stage	**1/2 MS**	**1%**	**45**	**Light green, relatively fewer and large**
	1/2 MS	2%	45	Light green, but too many and small
	1/2 MS	3%	45	Dark green, but too many and small
	MS	1%	45	Large, but usually vitrified
	MS	2%	45	Dark green, but too many and small
	MS	3%	45	Dark green, but too many and small
Shoot Elongation Stage	1/2 MS	1%	20	Abnormal with curly leaves
	**1/2 MS**	**2%**	**20**	**Well-developed leaves and root systems**
	1/2 MS	3%	20	Grow very slowly
	MS	1%	20	Grow very slowly
	MS	2%	20	Grow relatively slowly with curly leaves
	MS	3%	20	Abnormal with curly leaves

The optimal shoot induction media are highlighted by bold letters.

**Table 4:** Improvement of the transformation system of *C*. *pumila* in this study.

Parameters	Before Optimization [27]	After Optimization (This Study)
Explant orientation	Random	Adaxial leaf surfaces upward
*Agrobacterium* strain	LBA4404	EHA105, GV3101, LBA4404
AS concentration	150 mg/L (764 μM)	50 μM
OD600 value	0.6	0.4–0.6
Co-culture condition	2 days, room temperature	3 days, 26 °C;
Induction medium and duration	MS, 3% sucrose, 0.5 mg/L 6-BA, 0.1 mg/L NAA, 20 mg/L hygromycin, and 300 mg/L carbenicillin for 7–8 weeks	1/2 MS, 1% sucrose, 0.5 mg/L 6-BA, 0.1 mg/L NAA, 10 mg/L hygromycin, and 150 mg/L cefotaxime for 3–4 weeks
Selection medium and duration	MS, 3% sucrose, 0.5 mg/L 6-BA, 0.1 mg/L NAA, 20 mg/L hygromycin, and 300 mg/L carbenicillin before excised from explants	1/2 MS, 2% sucrose, and 10 mg/L hygromycin before transplanted to pots and cultured in greenhouse
Assessment way of the transformation system	Induction frequency	Transformation rate

## Supplementary Materials

The followings are available online at https://www.mdpi.com/article/10.3390/ijms22094544/s1. Figure S1: Effects of MS strength and sucrose concentration on *Chirita pumila* shoot regeneration and seedling elongation. Figure S2: The optimal phosphinothricin (PPT) concentration for selecting positive *Chirita pumila* transgenic lines. Figure S3: Expression analysis of *CpCYC* gene in wild type (WT) and *RNAi:CpCYC* transgenic flowers using quantitative real-time PCR. Figure S4: Varying degrees of actinomorphic flowers produced by T1 transgenic plants carrying *RNAi:CpCYC*. Figure S5: The diagram showing the construction of the RNAi:CpCYC vector. Table S1: Effect of different media on seed germination. Table S2: Measurement of the optimal bactericidal antibiotics for *Agrobacterium* elimination. Table S3: Effect of acetosyringone (AS) concentration on transformation rate. Table S4: Effect of *Agrobacterium* strains on transformation rate. Table S5: Optimization of *Agrobacterium* infection condition for *C. pumila* leaf explants. Table S6: Effect of co-culture temperature and duration on transformation rate. Table S7: Segregation ratios of T2 generations from five representative T1 *RNAi:CpCYC* lines. Table S8: Taxa and GenBank accession numbers of *rbcl*, *matk* and *ndhf* sequences for phylogenetic analyses.
