# Supplementary material for: An Optimized Transformation System and Functional Test of CYC-Like TCP Gene CpCYC in Chirita pumila (Gesneriaceae)
Source: Int J Mol Sci. 2021 Apr 27;22(9):4544. doi: 10.3390/ijms22094544 (PMC8123712; doi:10.3390/ijms22094544)
Supplement: Supplementary file 1 [file ijms-22-04544-s001.zip › ijms-1166721-Supplementary.pdf]

International Journal of *Molecular Sciences*

MDPI

Article

An Optimized Transformation System and Functional Test of *CYC*-Like TCP Gene *CpCYC* in *Chirita pumila* (Gesneriaceae)

Jing Liu [1,†], Juan-Juan Wang [1,2,†], Jie Wu [1,2,†], Yang Wang [1,2], Qi Liu [1,2], Fang-Pu Liu [1,2], Xia Yang [1,*] and Yin-Zheng Wang[1, 2,*]

[1] State Key Laboratory of Systematic and Evolutionary Botany, Institute of Botany, Chinese Academy of Sciences, Beijing 100093, China; liujing2010@ibcas.ac.cn (J.L.); jwang2@mpipz.mpg.de (J.-J.W.); daisywujie@mail.tsinghua.edu.cn (J.W.); wangyang2017@ibcas.ac.cn (Y.W.); liuqi2017@ibcas.ac.cn (Q.L.); liufangpu@ibcas.ac.cn (F.-P.L.)

[2] University of Chinese Academy of Sciences, Beijing 101408, China

* Correspondence: yangxia@ibcas.ac.cn (X.Y.); wangyz@ibcas.ac.cn (Y.-Z.W.)

† These authors contributed equally to this article.

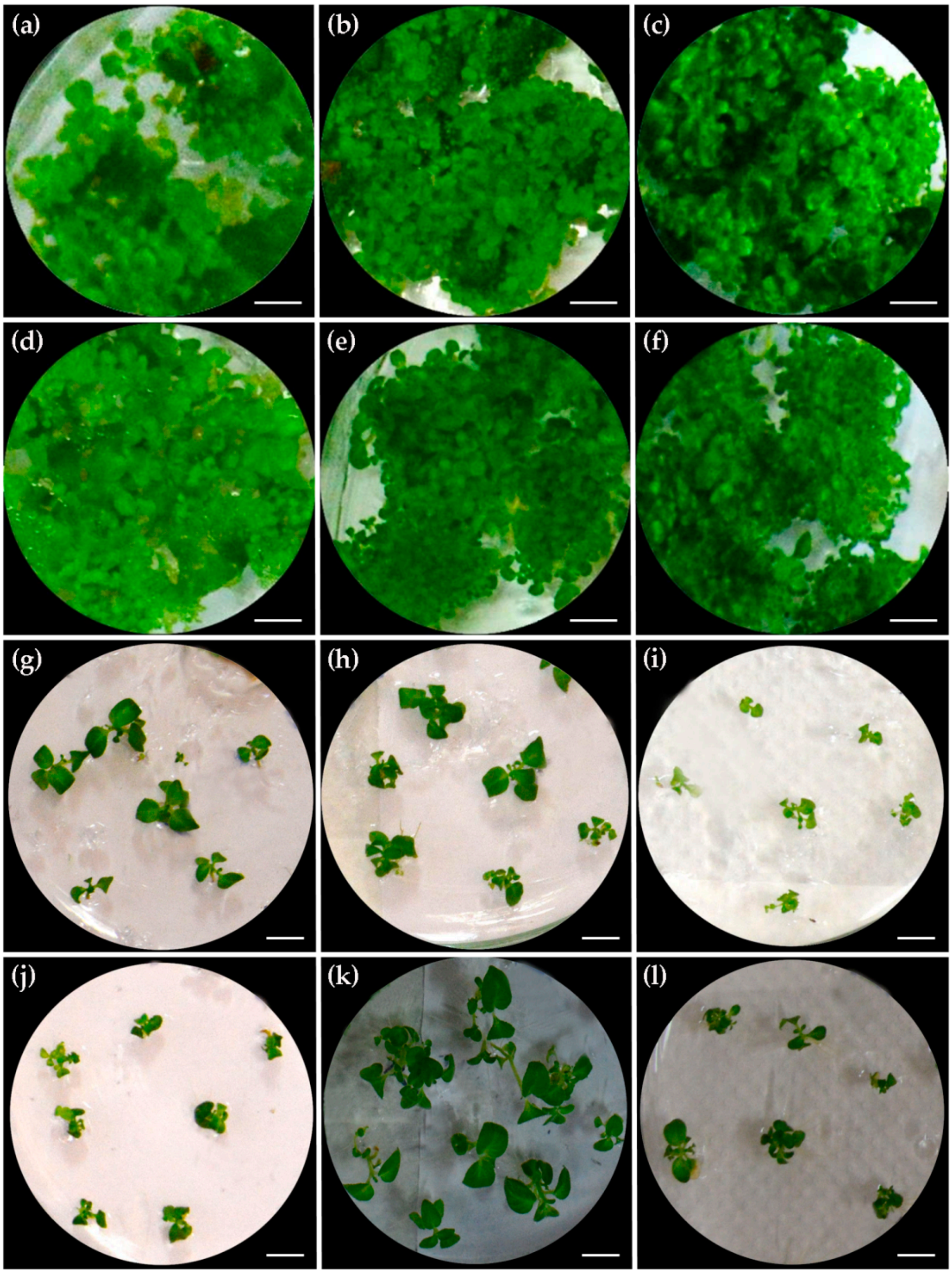

Figure S1. Effects of MS strength and sucrose concentration on *Chirita pumila* shoot regeneration and seedling elongation. (**a**) Adventitious shoots induced on MS medium containing 1% sucrose. (**b**) Adventitious shoots induced on MS medium containing 2% sucrose. (**c**) Adventitious shoots induced on MS medium containing 3% sucrose. (**d**) Adventitious shoots induced on 1/2 MS medium with 1% sucrose. (**e**) Adventitious shoots induced on 1/2 MS medium with 2% sucrose. (**f**) Adventitious shoots induced on 1/2 MS medium with 3% sucrose. (**g**) Seedlings grown on MS medium containing 1% sucrose. (**h**) Seedlings grown on MS medium containing 2% sucrose. (**i**) Seedlings grown on MS medium containing 3% sucrose. (**j**) Seedlings grown on 1/2 MS medium with 1% sucrose. (**k**) Seedlings grown on 1/2 MS medium with 2% sucrose. (**l**) Seedlings grown on 1/2 MS medium with 3% sucrose. Scale bars, 0.5cm in **a–f**; 1cm in **g–l**.

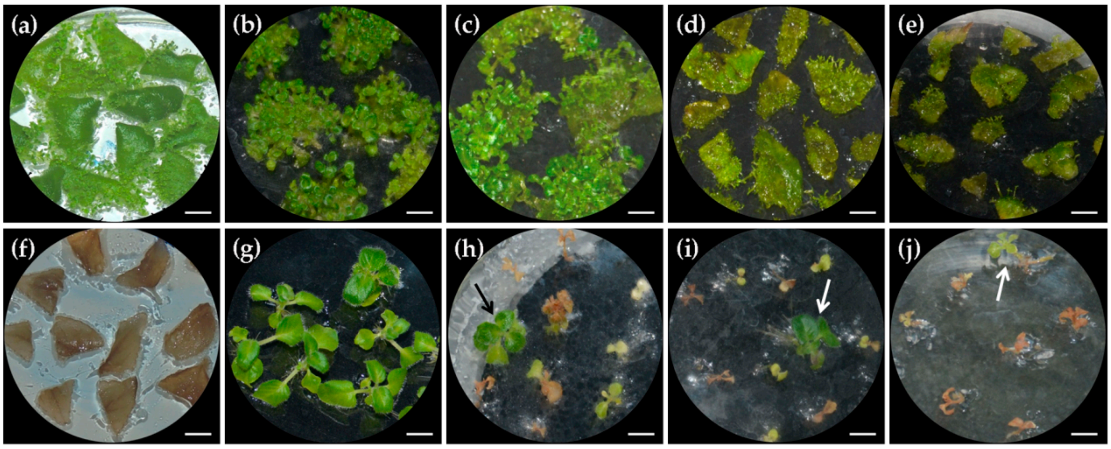

Figure S2. The optimal phosphinothricin (PPT) concentration for selecting positive *Chirita pumila* transgenic lines. (**a**) Wild-type explants cultured on medium without PPT. (**b–e**) The effect of PPT on bud induction of transfected explants. (**b**) 0 mg/L PPT; (**c**) 2 mg/L PPT; (**d**) 5 mg/L PPT; (**e**) 10 mg/L PPT. (**f**) Wild-type explants cultured on medium with 2 mg/L PPT. (**g–j**) The effect of PPT on the elongation of shoots from transfected explants. (**g**) 0 mg/L PPT; (**h**) 2 mg/L PPT; (**i**) 5 mg/L PPT; (**j**) 10 mg/L PPT. Arrows in **h–j** marked the positive transgenic seedlings confirmed by PCR. Scale bars, 0.5 cm.

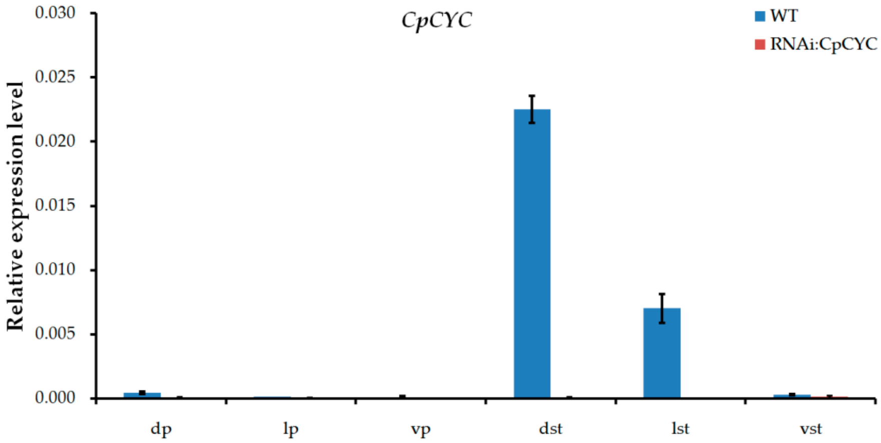

Figure S3. Expression analysis of *CpCYC* gene in wild-type (WT) and *RNAi:CpCYC* transgenic flowers using quantitative real-time PCR. *CpACTIN* was amplified as an internal reference. Data are mean ± SD from three biological replicates. dp/lp/vp, dorsal/lateral/ventral petals; dst/lst/vst, dorsal/lateral/ventral stamens.

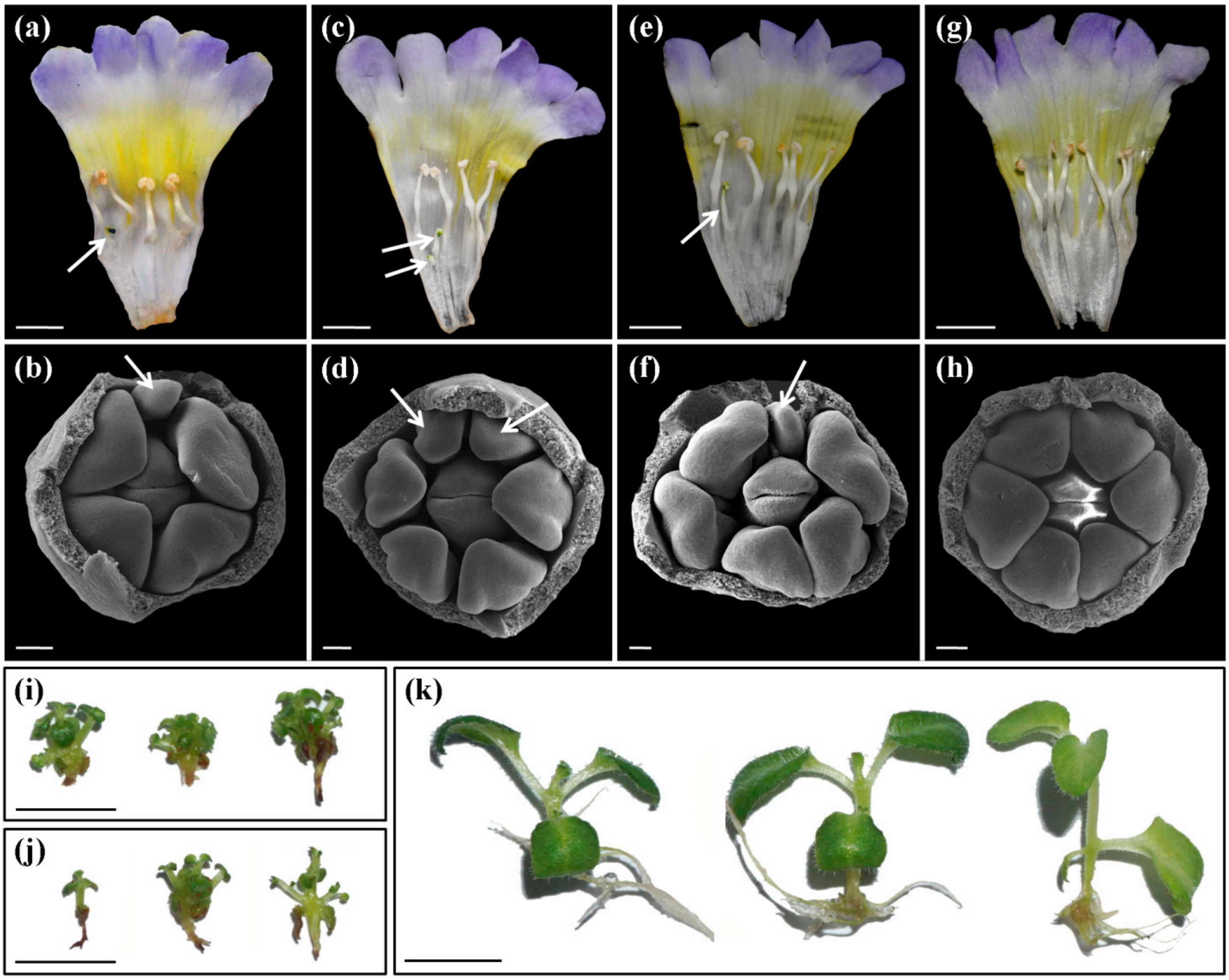

Figure S4. Varying degrees of actinomorphic flowers produced by T1 transgenic plants carrying *RNAi:CpCYC*. (**a**,**b**) A pentamerous flower with five identical petals and four fertile stamens. (**c**,**d**) A hexamerous flower with six identical petals and four fertile stamens. (**e**,**f**) A hexamerous flower with six identical petals and five fertile stamens. (**g**,**h**) A fully actinomorphic hexamerous flower with six identical petals and six fertile stamens. (**i**,**j**) The addition of 10 mg/L hygromysin inhibited the growth and development of wild-type (**i**) and negative T2 progenies of *RNAi:CpCYC* transgenic plants (**j**). (**k**) Positive T2 progenies of *RNAi:CpCYC* transgenic plants grew healthily with well-developed root systems under the selection pressure of 10 mg/L hygromysin. Scale bars, 0.5cm in **a**, **c**, **e**, **g**, **i**–**k**; 50μm in **b**, **d**, **f**, **h**.

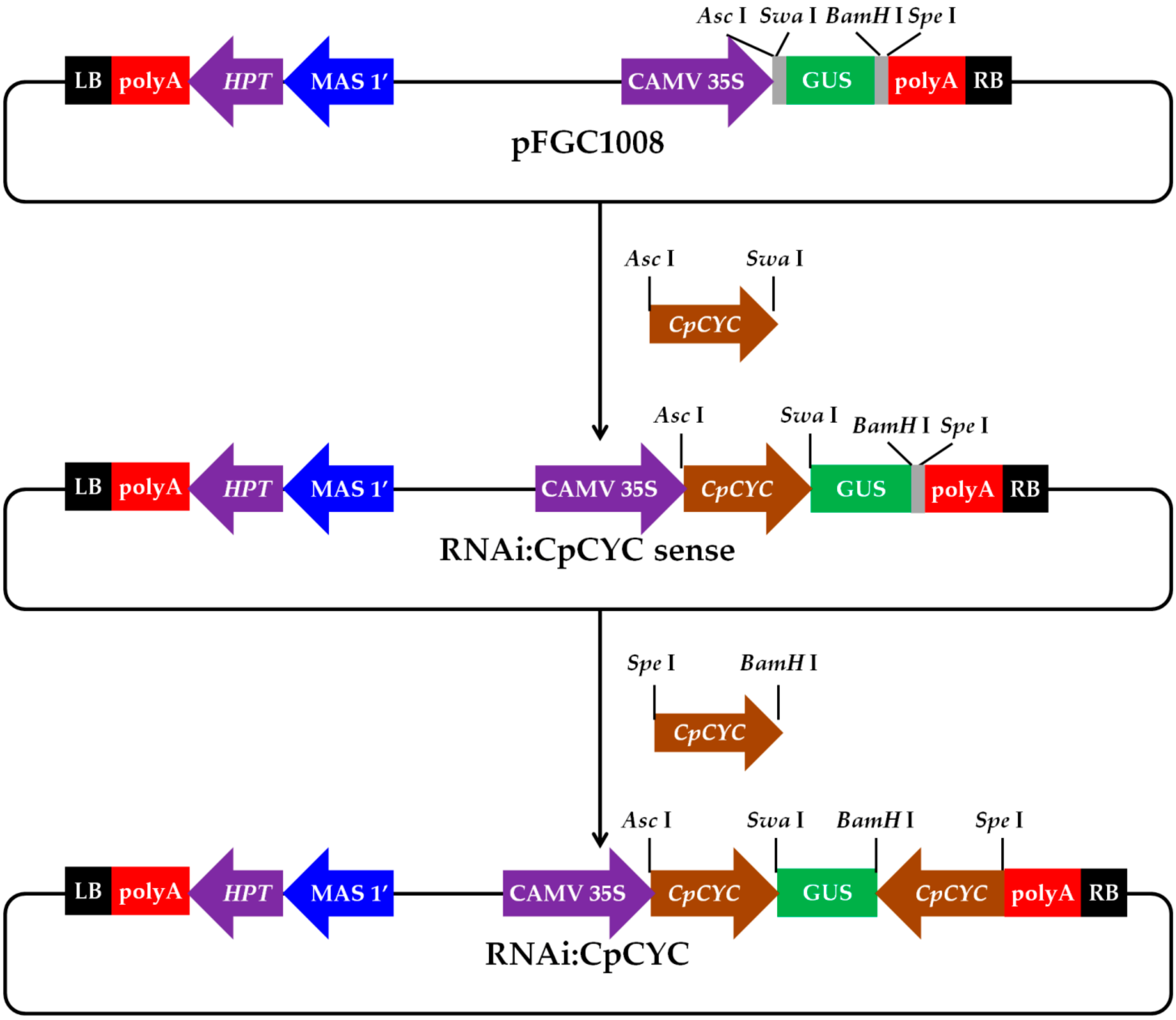

Figure S5. The diagram showing the construction of the RNAi:CpCYC vector. LB and RB mean left and right borders of the binary vector, respectively. *HPT*, the *hygromycin phosphotransferase* gene.

Table S1. Effect of different media on seed germination.

MS Salt Strength	Sucrose Concentration	Days for Seed Germination†
**1/2 MS**	**1%**	**7**
1/2 MS	2%	9
1/2 MS	3%	10
MS	1%	8
MS	2%	12
MS	3%	17

† Days for seed germination were defined as when green cotyledons can be clearly observed. The optimal medium for seed germination is highlighted by bold letters.

Table S2. Measurement of the optimal bactericidal antibiotics for *Agrobacterium* elimination.

Antibiotics	Concentration (mg/L)	Number of Explants	Number of Explants with Adventitious Shoots	Adventitious Shoot Induction Rate (%)
Carbenicillin	50	45	4	8.89
	50	45	3	6.67
	50	45	5	11.11
	100	45	23	51.11
	100	45	25	55.56
	100	45	21	46.67
	150	45	8	17.78
	150	45	10	22.22
	150	45	9	20.00
Cefotaxime	50	45	19	42.22
	50	45	22	48.89
	50	45	16	35.56
	100	45	21	46.67
	100	45	17	37.78
	100	45	17	37.78
	150	45	29	64.44
	150	45	26	57.78
	150	45	26	57.78
Timentin	50	45	10	22.22
	50	45	12	26.67
	50	45	8	17.78
	100	45	3	6.67
	100	45	6	13.33
	100	45	5	11.11
	150	45	12	26.67
	150	45	15	33.33
	150	45	9	20.00

Table S3. Effect of acetosyringone (AS) concentration on transformation rate.

AS Concentration (μM)	Number of Inoculated Explants	Number of Positive Explants	Transformation Rate (%)
0	45	0	0
	45	0	0
	45	0	0
50	45	5	11.11
	45	6	13.33
	45	5	11.11
100	45	3	6.67
	45	3	6.67
	45	4	8.89
200	45	0	0
	45	0	0
	45	0	0

Table S4. Effect of *Agrobacterium* strains on transformation rate.

Agrobacterium Strains	Number of Inoculated Explants	Number of Positive Explants	Transformation Rate (%)
LBA4404	39	18	46.15
	39	23	58.97
	40	24	60.00
GV3101	39	8	20.51
	39	5	12.82
	40	11	27.50
EHA105	39	4	10.26
	39	2	5.13
	40	6	15.00

Table S5. Optimization of *Agrobacterium* infection condition for *C. pumila* leaf explants.

OD600 Value	Infection Time (min)	Number of Explants	Number of Positive Explants	Transformation Rate (%)
0.4	10	30	0	0
	10	30	0	0
	10	30	0	0
	20	30	4	13.33
	20	30	3	10.00
	20	30	2	6.67
	30	30	2	6.67
	30	30	1	3.33
	30	30	3	10.00
0.6	10	30	0	0
	10	30	0	0
	10	30	0	0
	20	30	3	10.00
	20	30	5	16.67
	20	30	4	13.33
	30	30	0	0
	30	30	0	0
	30	30	0	0
0.8	10	30	1	33.33
	10	30	0	0
	10	30	0	0
	20	30	1	33.33
	20	30	0	0
	20	30	0	0
	30	30	0	0
	30	30	0	0
	30	30	0	0

Table S6 Effect of co-culture temperature and duration on transformation rate.

Co-culture Temperature (°C)	Co-culture Days	Number of Explants	Number of Positive Explants	Transformation Rate (%)
22	2	30	10	33.33
	2	30	12	40.00
	2	30	8	26.67
	3	30	5	16.67
	3	30	4	13.33
	3	30	6	20.00
24	2	30	5	16.67
	2	30	4	13.33
	2	30	3	10.00
	3	30	13	43.33
	3	30	10	33.33
	3	30	10	33.33
26	2	30	5	16.67
	2	30	7	23.33
	2	30	6	20.00
	3	30	15	50.00
	3	30	11	36.67
	3	30	10	33.33

Table S7. Segregation ratios of T2 generations from five representative T1 *RNAi:CpCYC* lines.

Line	Number of Hyg-resistant Seedlings	Number of Hyg-sensitive Seedlings	Mendelian Segregation Ratio
3	192	62	3.10 : 1
7	210	68	3.09 : 1
40	78	27	2.89 : 1
41	75	29	2.59 : 1
64	67	23	2.91 : 1

Table S8. Taxa and GenBank accession numbers of *rbcl*, *matk* and *ndhf* sequences for phylogenetic analyses.

**Taxa**	***rbcl***	***matk***	***ndhf***	**Chloroplast Genome**
Antirrhinum majus	GQ997015	AF375189	GQ996983	
Aquilegia caerulea	NC_041528.1:56936-58363	NC_041528.1:2423-3952	NC_041528.1:117630-119849	NC_041528
Arabidopsis thaliana	NC_000932.1:54958-56397	NC_000932.1:2056-3570	NC_000932.1:110398-112638	NC_000932
Aristolochia debilis	NC_036153.1:59349-60776	NC_036153.1:1944-3488	NC_036153.1:114840-117083	NC_036153
Calceolaria uniflora	unavailable	AJ580489	unavailable	unavailable
Capsella bursa-pastoris	NC_009270.1:54493-55932	NC_009270.1:2041-3621	NC_009270.1:110158-112398	NC_009270
Chirita pumila	KX527228	KX526643	KX526957	unavailable
Glycine max	NC_007942.1:5312-6739	NC_007942.1:2011-3528	NC_007942.1:124392-126644	NC_007942
Helianthus annuus	NC_007977.1:54553-56010	NC_007977.1:2065-3567	NC_007977.1:124170-126401	NC_007977
Lilium longiflorum	KC968977.1:52832-54274	KC968977.1:1709-3247	KC968977.1:108751-110973	KC968977
Lotus japonicus	NC_002694.1:5158-6585	NC_002694.1:2174-3700	NC_002694.1:107092-109335	NC_002694
Medicago truncatula	NC_003119.8:117623-119050	NC_003119.8:120387-121907	NC_003119.8:190-2424	NC_003119
Mimulus guttatus	NC_030212.1:55887-57341	NC_030212.1:2115-3653	NC_030212.1:109760-112009	NC_030212
Nicotiana benthamiana	unavailable	AB040014	unavailable	unavailable
Nigella damascena	NC_041537.1:57729-59156	NC_041537.1:1997-3529	NC_041537.1:112217-114430	NC_041537
Oryza sativa	NC_031333.1:54051-55484	NC_031333.1:1668-3203	NC_031333.1:101393-103597	NC_031333
Osmanthus fragrans	NC_042377.1:57266-58693	NC_042377.1:2122-3699	NC_042377.1:127396-129618	NC_042377
Petunia hybrida	NC_040178.1:57566-58999	NC_040178.1:2147-3670	NC_040178.1:112621-114843	NC_040178
Physalis floridana	NC_048514.1:57623-59056	NC_048514.1:1976-3505	NC_048514.1:112608-114830	NC_048514
Pisum sativum	NC_014057.1:4712-6139	NC_014057.1:1889-3418	NC_014057.1:119721-121967	NC_014057
Populus trichocarpa	NC_009143.1:55716-57143	NC_009143.1:1981-3513	NC_009143.1:112891-115173	NC_009143
Rehmannia glutinosa	NC_034308.1:55917-57350	NC_034308.1:2137-3672	NC_034308.1:110398-112620	NC_034308
Rosa hybrida	NC_044126.1:57054-58481	NC_044126.1:2108-3619	NC_044126.1:112272-114512	NC_044126
Senecio vulgaris	NC_046693.1:54435-55892	NC_046693.1:1959-3470	NC_046693.1:123761-125986	NC_046693
Sesamum indicum	NC_016433.2:56251-57684	NC_016433.2:1972-3501	NC_016433.2:110382-112637	NC_016433
Sinningia speciosa	unavailable	JX196113	AY364309	unavailable
Solanum lycopersicum	NC_007898.3:56683-58116	NC_007898.3:2124-3653	NC_007898.3:111505-113718	NC_007898
Vitis vinifera	NC_007957.1:59436-60863	NC_007957.1:2016-3524	NC_007957.1:59436-60863	NC_007957
